# PPARγ-dependent remodeling of translational machinery in adipose progenitors is impaired in obesity

**DOI:** 10.1016/j.celrep.2024.114945

**Published:** 2024-11-22

**Authors:** Mirian Krystel De Siqueira, Gaoyan Li, Yutian Zhao, Siqi Wang, In Sook Ahn, Mikayla Tamboline, Andrew D. Hildreth, Jakeline Larios, Alejandro Schcolnik-Cabrera, Zaynab Nouhi, Zhengyi Zhang, Marcus J. Tol, Vijaya Pandey, Shili Xu, Timothy E. O’Sullivan, Julia J. Mack, Peter Tontonoz, Tamer Sallam, James A. Wohlschlegel, Laura Hulea, Xinshu Xiao, Xia Yang, Claudio J. Villanueva

**Affiliations:** 1 Department of Integrative Biology and Physiology, University of California, Los Angeles, Los Angeles, CA 90095, USA; 2 CAS Key Laboratory of Computational Biology, Shanghai Institute of Nutrition and Health, Chinese Academy of Sciences, Shanghai 200031, China; 3 Crump Institute for Molecular Imaging, University of California, Los Angeles, Los Angeles, CA 90025, USA; 4 Department of Molecular and Medical Pharmacology, University of California, Los Angeles, Los Angeles, CA 90025, USA; 5 Department of Microbiology, Immunology, and Molecular Genetics, David Geffen School of Medicine, University of California, Los Angeles, Los Angeles, CA 90095, USA; 6 Molecular Biology Institute, University of California, Los Angeles, Los Angeles, CA 90095, USA; 7 Maisonneuve-Rosemont Hospital Research Centre, Montréal, QC H1T 2M4, Canada; 8 Département de Biochimie et Médecine Moléculaire, Université de Montréal, Montréal, QC H3C 3J7, Canada; 9 Département de Médecine, Université de Montréal, Montréal, QC H3C 3J7, Canada; 10 Department of Pathology and Laboratory Medicine, University of California, Los Angeles, Los Angeles, CA 90095, USA; 11 Department of Biological Chemistry, University of California, Los Angeles, Los Angeles, CA 90095, USA; 12 Jonsson Comprehensive Cancer Center, University of California, Los Angeles, Los Angeles, CA 90025, USA; 13 Department of Medicine, Division of Cardiology, Los Angeles, Los Angeles, CA 90095, USA; 14 These authors contributed equally; 15 Lead contact

## Abstract

Adipose tissue regulates energy homeostasis and metabolic function, but its adaptability is impaired in obesity. In this study, we investigate the impact of acute PPARγ agonist treatment in obese mice and find significant transcriptional remodeling of cells in the stromal vascular fraction (SVF). Using single-cell RNA sequencing, we profile the SVF of inguinal and epididymal adipose tissue of obese mice following rosiglitazone treatment and find an induction of ribosomal factors in both progenitor and preadipocyte populations, while expression of ribosomal factors is reduced with obesity. Notably, the expression of a subset of ribosomal factors is directly regulated by PPARγ. Polysome profiling of the epididymal SVF shows that rosiglitazone promotes translational selectivity of mRNAs that encode pathways involved in adipogenesis and lipid metabolism. Inhibition of translation using a eukaryotic translation initiation factor 4A (eIF4A) inhibitor is sufficient in blocking adipogenesis. Our findings shed light on how PPARγ agonists promote adipose tissue plasticity in obesity.

## In brief

De Siqueira et al. explore the effects of rosiglitazone on adipose stem cell plasticity, showing that PPARγ stimulates expression of ribosomal factors in adipocyte precursors. This leads to translational selectivity of mRNAs involved in lipid storage. They find ribosomal function to be a potential therapeutic target in treating metabolic disorders.

## Graphical Abstract

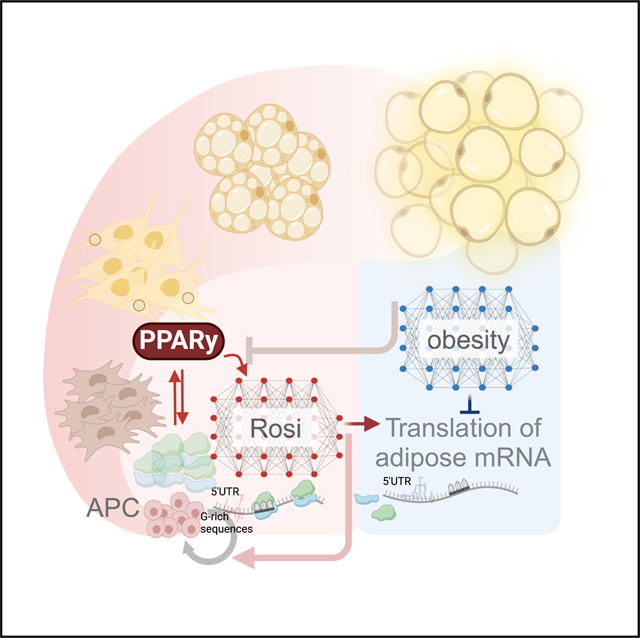

## INTRODUCTION

Obesity is a complex and multifactorial condition that results from an imbalance between energy intake and energy expenditure. Excess accumulation of body fat can negatively impact an individual’s physical, psychological, and social well-being as well as increasing the risk of developing several chronic diseases, such as cardiovascular disease, type 2 diabetes, and certain cancers. This complex interplay of adipose tissue adaptation and expansion during obesity can ultimately lead to maladaptive changes in its structure and function.^1,2^

While the maladaptation of the adipose tissue during obesity has been studied extensively,^3^ it is not clear how distinct cell types within adipose tissue are impacted. Recent single-cell and single-nucleus RNA-sequencing (RNA-seq) studies have uncovered significant heterogeneity and plasticity of adipose progenitor cells (APCs) in mice and humans.^4–17^ External factors such as β-adrenergic stimulation drive dynamic remodeling of APCs, in both brown adipose tissue (BAT) and inguinal white adipose tissue (iWAT),^11,15^ while obesity is characterized by a decline in the number of APCs and a shift toward a fibroinflammatory and less lipogenic profile.^4,9^

The impact of APCs on glucose metabolism was first reported using an inducible adipocyte-specific PPARγ-knockout system, where mice lacking PPARγ and treated with thiazolidinediones (TZDs—PPARγ agonists) showed improved glucose metabolism. This observation suggests that there are either indirect effects of TZDs or PPARγ targets outside of adipocytes.^18^ TZDs have been studied extensively, and one of them, rosiglitazone (Rosi), can improve insulin sensitivity through direct activation of PPARγ, which promotes lipogenesis and secretion of adiponectin, reduces inflammation in adipose tissue, and decreases hepatic and peripheral triglyceride levels.^19–24^ Clinical use of TZDs has shown potent insulin-sensitizing properties in individuals with type 2 diabetes, but their use has decreased in past years due to concerns about increasing cardiovascular risk.^25,26^ Interestingly, better-controlled studies, such as the RECORD clinical trial that was designed to investigate cardiovascular outcomes, concluded that there was no increased risk of heart attack or death. Compared with standard glucose-lowering drugs, Rosi does not increase the risk of overall cardiovascular morbidity or mortality.^27^ Recent studies are in favor of individualized treatment, for instance, including TZDs as a combination therapy in patients with no cardiovascular disease history, since these medications (e.g., pioglitazone) are highly effective in decreasing nonalcoholic steatohepatitis and fibrosis, and overall TZDs are more affordable compared to other therapeutics.^28^ Thus, tailoring combination therapy to meet individual patient needs appears to be the future standard of care for type 2 diabetes mellitus (T2DM) patients.

Given the renewed clinical interest in TZDs, we aimed to understand not only the transcriptional landscape regulated by Rosi in the adipose tissue but also the translational regulation of these messenger RNA (mRNA) networks. Rosi has been shown to increase the mammalian target of rapamycin complex 1 (mTORC1) activity, and deficiency of mTORC1 blocks the Rosi effect on transcriptional remodeling in the adipose tissue.^29,30^ One of the downstream targets of mTORC1 activation is the phosphorylation and, consequently, dissociation of eukaryotic translation initiation factor 4E-binding protein 1 (eIF-4EBP1) from the cap-dependent eukaryotic translation initiation factor 4E (eIF4E).^31–35^ As a consequence, eIF4E can then be recruited to the translation initiation complex eIF4F. In addition, mTORC1 is known to phosphorylate S6K, which in turn phosphorylates several components of the translation machinery (e.g., S6 and eIF4B).^36^ Furthermore, recent studies have implicated the expression of eukaryotic initiation factors, such as eIF2A, eIF6, and eIF4E, with several metabolic phenotypes. Full body knockouts for these initiation factors alter the translational landscape and the regulation of metabolic processes that impact insulin sensitivity and lipid metabolism.^37–39^ However, how the translational machinery is regulated in the context of obesity and TZD-driven remodeling is still not fully understood.

Here, we used single-cell RNA-seq (scRNA-seq) to investigate the impact of acute PPARγ agonist treatment in obese mice in both the inguinal and the epididymal adipose stromal vascular fraction (SVF). We demonstrated that Rosi remodels the molecular landscape in both depots and restores the obese transcriptional profile to what is observed in lean mice. In addition to developing a single-cell transcriptome atlas of leptin-deficient mice (ob/ob), and their response to Rosi in each cell type, we identified a role for PPARγ agonists in regulating translation. We have shown that Rosi increased the expression of ribosomal genes in the APCs and that this remodeling is induced by PPARγ directly binding to enhancers in close proximity to these genes. Moreover, heavily translating ribosomes (polysomes) were occupied not only by fat-specific PPARγ targets but also by a distinct selection of transcripts containing specific G-rich motifs in their 5′ untranslated region (5′ UTR) after Rosi treatment. In conclusion, we propose that this remodeling drives a translation selectivity in the polysomes of the SVF characterizing a specialized adipose tissue translation response to Rosi to drive adipogenesis and maintain adipose homeostasis.

## RESULTS

### Acute Rosi treatment confers improvement in glucose tolerance and structural remodeling of adipose tissue

**Figure 1.**
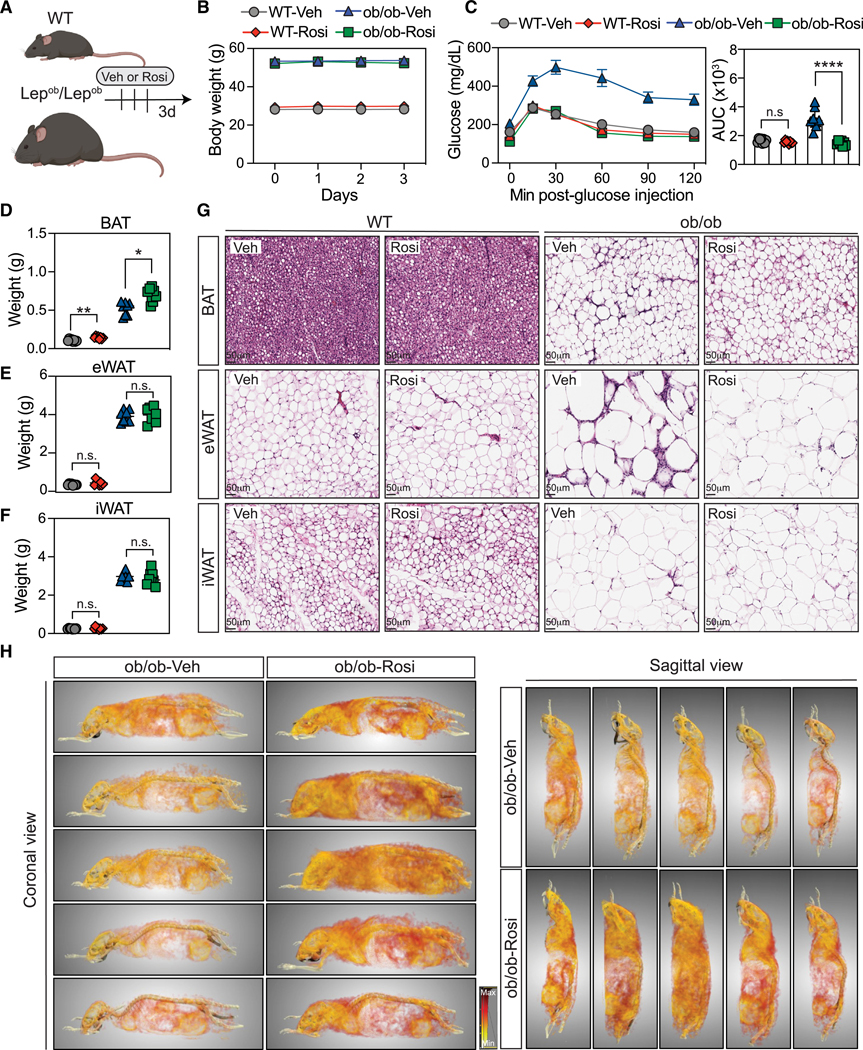
Adipose tissue remodeling after acute rosiglitazone treatment (A) Schematic experimental design: lean (WT) and obese (ob/ob) mice were gavaged with either vehicle (Veh) or rosiglitazone (Rosi) (30 mg/kg) for 3 days. (B) Total body weight after treatment regimen. (C) Intraperitoneal glucose tolerance test with 1 g/kg of glucose. (D–F) Adipose tissue weight: brown adipose tissue (BAT), epididymal white adipose tissue (eWAT), and inguinal white adipose tissue (iWAT). (G) Histological analysis of BAT, eWAT, and iWAT (hematoxylin and eosin staining). (H) Visualization of adipose tissue by PET-CT 1 h after administration of [^18^F]-FDG (100 mCi) with 1 g/kg of glucose. Data represent the mean ± SEM (*n* = 8 mice per group). GraphPad (GP) *p* value style: *p = 0.0332, **p < 0.0021, ***p < 0.0002, and ****p < 0.0001 by one-way ANOVA, multiple comparisons followed by Tukey’s *post hoc* test, or (C) two-way ANOVA followed by Bonferroni’s *post hoc* test.

To evaluate the impact of acute PPARγ agonist treatment, we treated wild-type (WT) C57BL/6 lean mice and obese *Lep*^*ob*^*/Lep*^*ob*^ (ob/ob) mice with either Rosi or vehicle (Veh) by oral gavage for 3 days (Figure 1A). Lean or ob/ob mice treated with vehicle (WT-Veh) or Rosi (WT-Rosi) had similar body weights after treatment (Figure 1B). In contrast, acute treatment with Rosi improved the glucose excursion curves of ob/ob mice compared to ob/ob Veh controls (Figure 1C), while Rosi did not improve the glucose tolerance test of (WT) lean mice. Thus, Rosi was able to normalize the glucose tolerance test of ob/ob mice within 3 days of acute treatment in the absence of changes in body weight.

To assess whether acute treatment with Rosi leads to morphological changes in adipose tissue and liver, we analyzed histological sections of WT and ob/ob mice. WT-Rosi mice presented no hepatic histological changes but a minor increase in total mass (Figures S1A and S1B). In contrast, the liver of ob/ob-Rosi mice showed a decrease in lipid accumulation (Figure S1A) characterized by a reduction in lipid droplet numbers and size (Figure S1C). Next, we examined whether Rosi impacted the weight and histology of brown adipose tissue (BAT), epididymal white adipose tissue (eWAT), and inguinal white adipose tissue (iWAT). We found that BAT mass increased with Rosi treatment in both lean and obese mice (Figure 1D), while the weights of eWAT and iWAT were similar compared between vehicle and Rosi (Figures 1E and 1F). Notably, histology of the eWAT from ob/ob mice treated with Rosi showed a decrease in crown-like structures with no major alterations in the iWAT or BAT in either obese or lean mice (Figure 1G).

To understand which tissues contributed to the improvements in glucose tolerance test in response to Rosi, we measured glucose uptake in ob/ob mice using an adapted conventional glucose tolerance test by mixing a bolus of glucose with [^18^F]fluorodeoxyglucose ([^18^F]-FDG). After 1 h of conscious glucose uptake, imaging was completed by positron emission tomography-computed tomography (PET-CT) scans (Figures 1H and S1D). ob/ob-Rosi mice presented an increase in glucose uptake in the brain, heart, liver, muscle, and adipose tissue (axillary) and a trend in the BAT compared to ob/ob-Veh mice (Figures S1E and S1F). To visualize glucose uptake in adipose tissue depots more clearly, we excluded regions of high [^18^F]-FDG uptake (e.g., heart and bladder) using the software Dragonfly (Object Research Systems). Remarkably, ob/ob-Rosi mice had greater glucose uptake in the adipose tissues compared to ob/ob-Veh mice (Figure 1H; Videos S1 and S2). To evaluate whether Rosi treatment induced expression of beige/brown adipocyte markers within this period, we measured the expression of thermogenic genes in both SVF and mature adipocytes (mADs). We observed upregulation of the thermogenic program in the mADs of iWAT/eWAT in ob/ob-Rosi mice compared to controls. However, only a minor change was detected in the SVF (Figure S1G).

### Single-cell RNA sequencing uncovers stromal vascular cell remodeling after PPARγ agonist treatment

**Figure 2.**
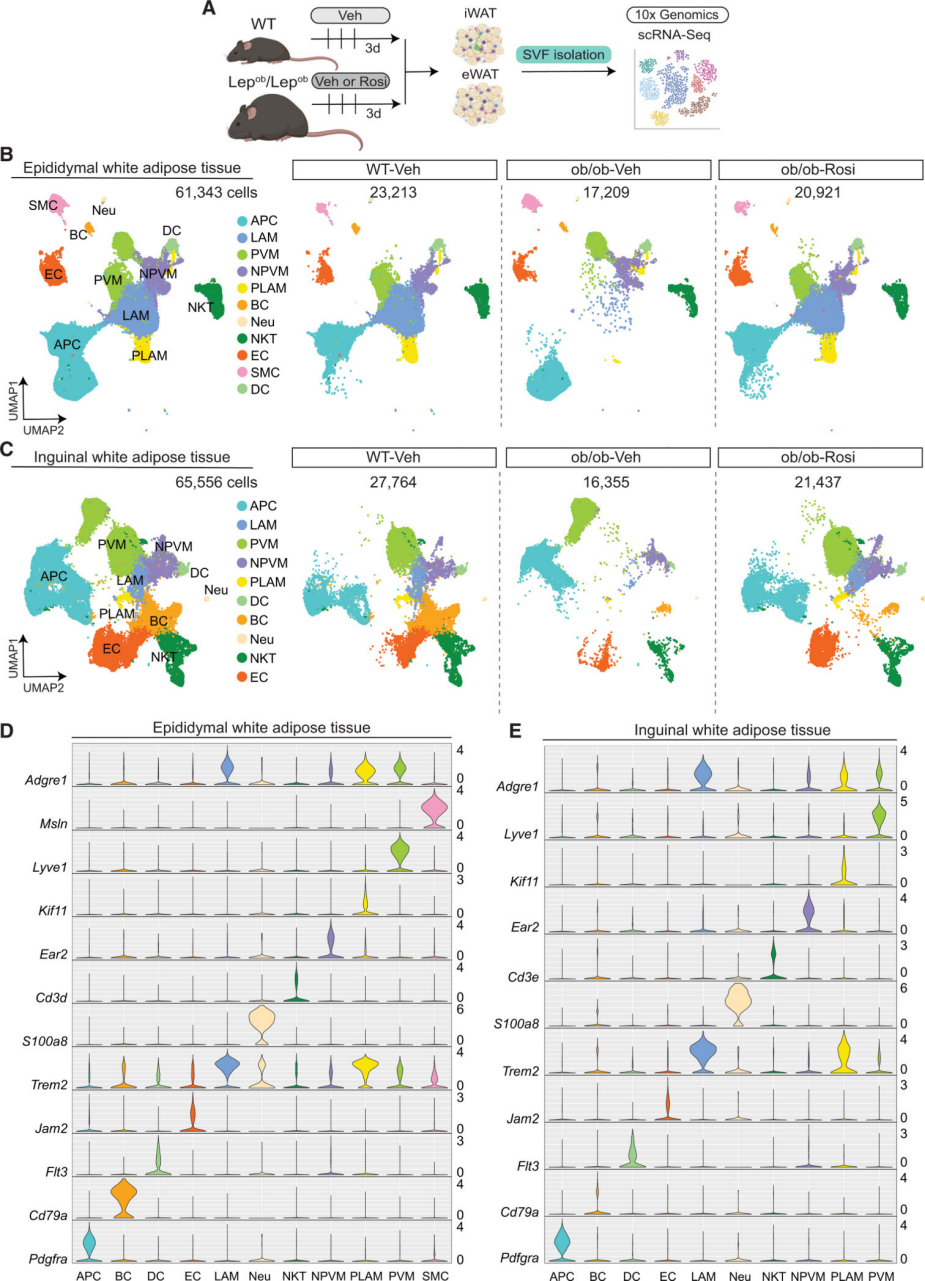
PPARγ-driven regulation of adipose tissue heterogeneity (A) Schematic overview of scRNA-seq: lean C57BL/6J (WT) and obese (ob/ob) mice were treated with either Veh or Rosi for 3 days (30 mg/kg). scRNA-seq was conducted on the stromal vascular fraction extracted from epididymal white adipose tissue (eWAT) and inguinal white adipose tissue (iWAT) separately. (B and C) Uniform manifold approximation and projection (UMAP) plots illustrate the cell clusters among 61,343 eWAT cells and 65,556 iWAT cells. The right three plots separately represent cells from WT-Veh, ob/ob-Veh, and ob/ob-Rosi. Each colored dot signifies a cell, with distinct colors indicating various cell types. The Louvain algorithm was utilized to determine cell clusters. (D and E) Cluster-specific expression of known cell markers.

The observation that the mass of eWAT and iWAT of ob/ob mice did not increase in response to Rosi, despite a reduction in adipocyte size, suggests that acute Rosi treatment induces the formation of new adipocytes. Consequently, it is likely that TZDs are initiating the differentiation of adipocyte progenitors into nascent adipocytes, thereby promoting the remodeling of SVF. To further investigate how Rosi treatment influences SVF in adipose tissue, we employed scRNA-seq in both eWAT and iWAT. Since the major Rosi responses were observed in obese mice, we treated ob/ob mice with either Veh or Rosi for 3 days. In addition, we included a WT-Veh group to compare the baseline of lean mice (Figure 2A). Briefly, datasets from both eWAT and iWAT were separately processed through the 10× Genomics Cell Ranger version 3.0.2 single-cell software to perform sample demultiplexing, sequence alignment to mouse genome mm10, quality control assessment, filtering, and unique molecular identifier (UMI) counting to generate gene count matrices (Figures S2A and S2B). We identified the major cell types described to be present in the adipose tissue: smooth muscle cells (SMCs), neutrophils, B cell lymphocytes (BCs), endothelial cells (ECs), adipocyte progenitor cells (APCs), natural killer T cells (NKTs), dendritic cells (DCs), perivascular macrophages (PVMs), lipid-associated macrophages (LAMs), non-perivascular-like macrophages (NPVMs), and proliferating-lipid-associated macrophages (PLAMs) (Figures 2B–2E). Visualization of the cell clusters using t-distributed stochastic neighbor embedding (t-SNE) plots of the individual mice showed high reproducibility within groups (Figures S2C and S2D). Remarkably, ob/ob-Rosi samples from both eWAT and iWAT presented a shift in the cell clusters away from those in the ob/ob-Veh group to resemble the microenvironment in the lean WT-Veh mice (Figures 2B and 2C).

### Rosi treatment leads to a major shift in macrophage subpopulations

Despite the presence of crown-like structures in the histology of the ob/ob-Veh mice (Figure 1G), we found few LAMs by scRNA-seq in the ob/ob-Veh samples (eWAT and iWAT) (Figures 2B and 2C). We hypothesized that LAMs floated with mADs because of their high lipid content and the gentle dissociation protocol used for scRNA-seq (see STAR Methods). To test this, we collected the top floating layer after eWAT/iWAT enzymatic digestion, plated cells overnight, and then performed confocal imaging. We found numerous F4/80-positive cells within the mAD fraction in both eWAT and iWAT of ob/ob-Veh mice (Figure S3A). A few of these macrophages (Macs) were in the process of phagocytosing apoptotic adipocytes, which also showed Plin1 staining, a lipid droplet marker found in adipocytes. In contrast, ob/ob-Rosi samples showed a decrease in the number of macrophages, as well as drastic morphological differences in macrophages, likely reflecting a different state of activation with fewer classically activated cells (Figure S3A). The effect of Rosi on macrophages switching from an inflammatory to an anti-inflammatory profile has been previously described,^40–42^ and PPARγ activation directs an alternative-activation profile by the interleukin-4 (IL-4)-PPARγ-STAT6 pathway. To better understand the changes in macrophage composition in response to Rosi, we performed flow cytometry to quantify macrophages, inflammatory macrophages (IMs), LAMs, and PVMs (Figure S3B). We found that Rosi treatment decreased the number of IMs and LAMs in both eWAT and iWAT, with a stronger response in the eWAT (Figures S3C–S3F).

### Impact of PPARγ agonist treatment on adipocyte progenitor cells

**Figure 3.**
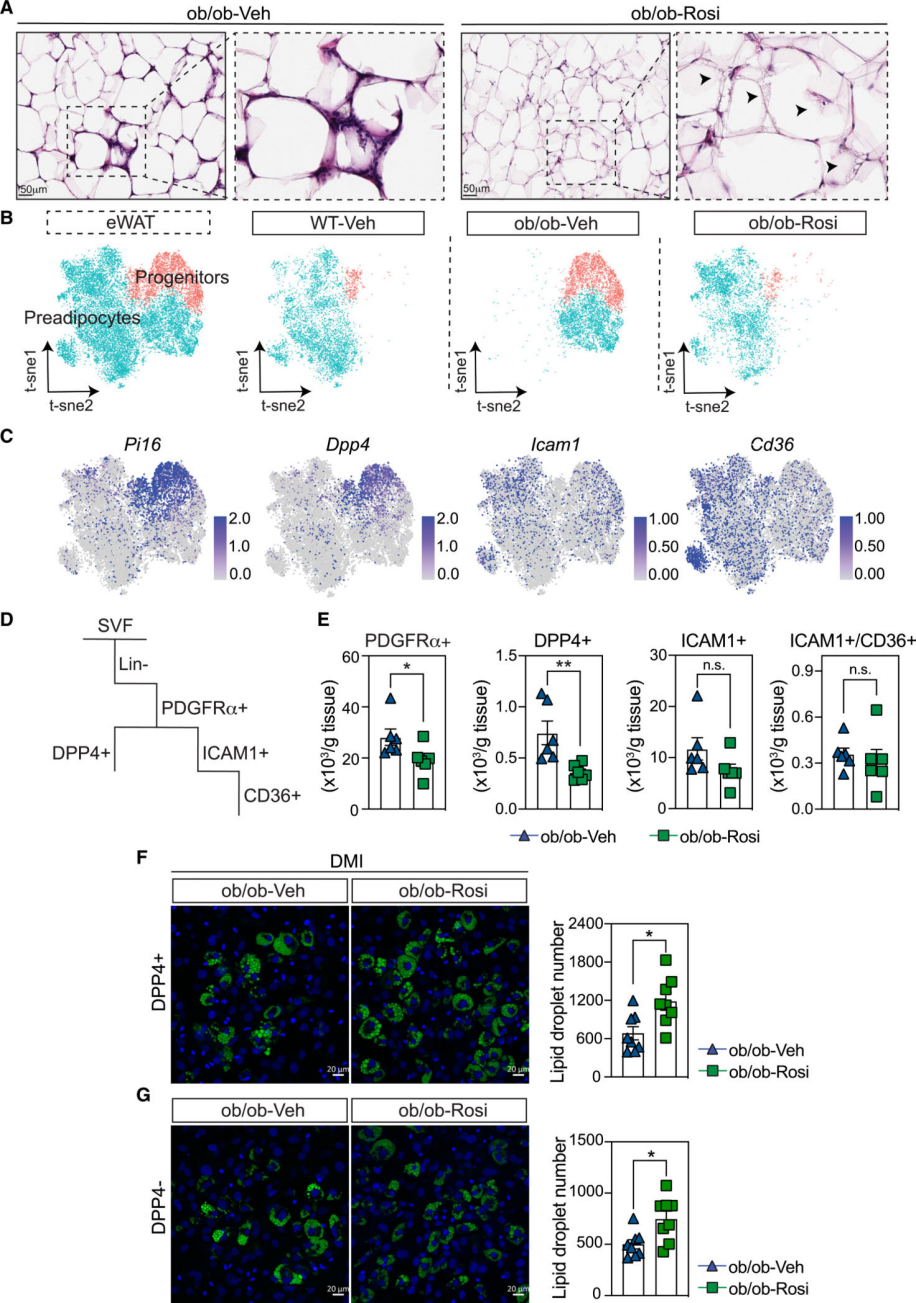
Remodeling of epididymal adipocyte precursor cells in response to PPARγ agonist (A) Histological analysis of epididymal white adipose tissue (hematoxylin and eosin stain) from either ob/ob-Veh- or ob/ob-Rosi-treated mice. (B) t-distributed stochastic neighbor embedding (t-SNE) plot illustrates two subclusters of adipocyte precursor cells in the eWAT: progenitors and preadipocytes. The right three plots separately represent cells from WT-Veh, ob/ob-Veh, and ob/ob-Rosi. Each color-coded dot represents a cell, with progenitors being represented by red and preadipocytes by cyan. The Louvain algorithm was utilized to determine cell clusters. (C) Individual gene t-SNE plots showing the expression and distribution of representative marker genes: *Pi16* and *Dpp4* for progenitors, *Icam1* and *Cd36* for preadipocytes. (D) Gate strategy to characterize progenitors and preadipocytes. (E) Absolute numbers of progenitors and preadipocytes from ob/ob-Veh and ob/ob-Rosi mice. (F and G) Images of confocal microscopy of sorted lineage-negative (CD45^-^, CD31^-^), PDGFRα^+^, DPP4^+^, and DPP4^-^ cells differentiated for 4 days with DMI medium, with DAPI (nuclei, blue) and LipidTox (neutral lipids, green) staining. Data represent the mean ± SEM (*n* = 6 mice per group). Confocal images: four wells per condition, two representative images per well were acquired. GraphPad (GP) *p* value style: *p = 0.0332, **p < 0.0021, and ***p < 0.0002 by two-tailed Student’s t test.

Further evaluation of histology showed that both eWAT (Figure 3) and iWAT (Figure S4) have emerging multilocular cells that are indicative of newly forming adipocytes in ob/ob-Rosi mice (Figures 3A and S4A, arrowheads). To evaluate whether acute Rosi treatment could impact the adipocyte progenitor populations that give rise to adipocytes, we focused on the cluster of APCs that were then subclustered into progenitors and preadipocytes (Figures 3B and S4B). Interestingly, Rosi treatment of ob/ob mice remodeled the clusters of progenitors and preadipocytes to states that were transcriptionally similar to those observed in lean mice (Figures 3B and S4B). To confirm the progenitor and preadipocyte identities, we checked the expression of classical markers of progenitors (*Pi16* and *Dpp4*) and preadipocytes (*Icam1* and *Cd36*). eWAT presented a very clear and distinguishing expression of progenitor markers and a more diffused expression of preadipocyte markers, potentially exemplifying a gradient of commitment to the adipogenic lineage (Figure 3C). In contrast, iWAT had very clear separation between progenitors and preadipocytes (Figure S4C). Next, we made several comparisons to understand how our APC annotations compared with previously published single-cell analyses to validate our progenitor and preadipocyte populations (Figures S5A and S5B). Our dataset was proven to be similar to previously characterized APCs in eWAT and iWAT^43,44^ and single-nucleus RNA-seq from high-fat-fed mice.^9^

To determine whether Rosi impacts the total number of ob/ob progenitors and preadipocytes in WAT, we performed flow cytometry (Figures 3D and S4D). Since both eWAT and iWAT showed high expression of *Pdgfrα* (Figures S5C and S5D), we defined our gating strategy as follows: lineage exclusion or Lin^-^ (CD31^-^, CD45^-^) and PDGFRα^+^, followed by the isolation of progenitors (DPP4^+^) and preadipocytes (ICAM1^+^ and ICAM1^+^/CD36^+^) (Figure S5E). In both tissues, we observed a reduction in both frequency and absolute numbers of the less committed populations (PDGFRα^+^ and DPP4^+^) in the ob/ob-Rosi mice compared to ob/ob-Veh (Figures 3E, S4E, S5F, and S5G). Interestingly, we also observed a reduction in the preadipocyte ICAM1^+^ cells in the eWAT at frequency level (Figure S5F) and in iWAT at both absolute number and frequency levels in ob/ob-Rosi mice (Figures S4E and S5G). We then hypothesized that such reduction could reflect more differentiation of those progenitors/preadipocytes into fully mature fat cells. We subsequently gated those preadipocytes with CD36^+^ as a marker of a more differentiated preadipocyte. Even though the eWAT ICAM1^+^ cells did not show a significant increase in the number of ICAM^+^/CD36^+^ cells after Rosi treatment, we did observe more ICAM^+^/CD36^+^ iWAT cells in ob/ob mice treated with Rosi (Figures 3E and S4E). This response in iWAT is consistent with previous observations showing a more robust response to Rosi in subcutaneous adipose tissue.^23^

To test the differentiation potential of APCs after Rosi treatment, we sorted cells from ob/ob mice acutely treated for 3 days with vehicle or Rosi and plated them on a tissue culture plate. Progenitors were defined as Lin^-^, PDGFRα^+^, DPP4^+^, while preadipocytes were defined as Lin^-^, PDGFRα^+^, DPP4^-^ (Figures 3F, 3G, S4F, and S4G). eWAT DPP4^+^ and DPP4^-^ cells from ob/ob-Rosi mice both showed an increase in adipocyte differentiation after 4 days in complete differentiation medium (DMI), suggesting an *ex vivo* advantage in those cells toward the adipogenic lineage (Figures 3F and 3G). iWAT DPP4^+^ and DPP4^-^ cells from ob/ob-Rosi mice, however, showed a very high adipogenic capacity in the same medium, making it hard to precisely quantify lipid droplet number and size (Figures S6A and S6B). Even though both populations of iWAT DPP4^+^ and DPP4^-^ presented a trend toward more differentiation and smaller lipid droplets in the ob/ob-Rosi samples compared to ob/ob-Veh mice, we decided to complete a similar experiment in two additional conditions: (1) dexamethasone and insulin medium (DI) and (2) minimal medium with insulin. In a similar way, iWAT DPP4^+^ and DPP4^-^ cells from ob/ob-Rosi mice treated with DI presented a trend towards more adipogenic differentiation compared to ob/ob-Veh cells (Figures S6C and S6D). Indeed, in minimal medium, iWAT DPP4^+^ and DPP4^-^ cells from ob/ob-Rosi mice presented a significant increase in adipocyte differentiation in both populations compared to ob/ob-Veh mice (Figures S4F and S4G).

### Rosi-dependent remodeling of specific gene networks in adipocyte progenitor cells

**Figure 4.**
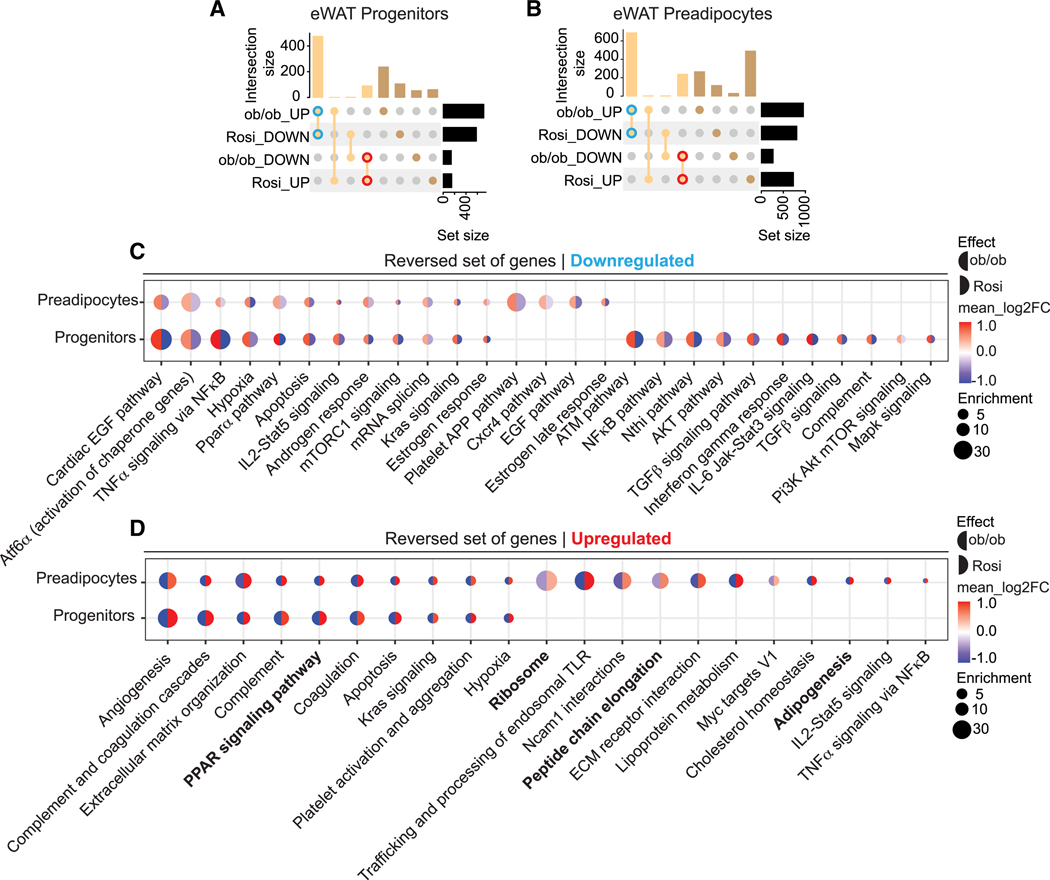
Comparison of differently expressed genes and enriched pathways in response to obesity and rosiglitazone treatment in the eWAT (A and B) UpSet plots illustrating the intersection of differentially expressed genes (DEGs) from eWAT progenitors (A) and eWAT preadipocytes (B), all at Benjamini-Hochberg adjusted *p* < 0.05. The four categories include upregulated DEGs in obese mice compared to lean mice (ob/ob_UP), downregulated DEGs in obese mice compared to lean mice (ob/ob_DOWN), upregulated DEGs in response to Rosi treatment compared to ob/ob-Veh (Rosi_UP), and downregulated DEGs in response to Rosi treatment compared to ob/ob-Veh (Rosi_DOWN) (see STAR Methods for details). (C and D) Dot plots illustrate the top enriched pathways in response to Rosi treatment, which acts to reverse the effects of obesity. All pathways displayed meet the cutoff for statistical significance at Benjamini-Hochberg-adjusted *p* < 0.05 (see STAR Methods for details). (C) Pathway enrichment from DEGs that are upregulated in eWAT obese mice and downregulated following Rosi treatment. (D) Represented pathways enriched from DEGs that are downregulated in eWAT obese mice and upregulated following Rosi treatment.

Next, we aimed to identify transcripts in progenitors/preadipocytes that correlate with the improvements in glucose metabolism observed in ob/ob mice treated with Rosi. Specifically, we focused on transcripts that are upregulated in ob/ob mice treated with Rosi and transcripts that are downregulated in ob/ob-Veh mice compared to lean mice (Figures 4 and S7). Our findings show that Rosi treatment mainly decreased the upregulated obesity-associated genes in the eWAT progenitor/preadipocyte cells (Figures 4A and 4B). eWAT preadipocytes showed a greater overlap in genes upregulated by obesity and downregulated by Rosi than the progenitor cells (Figures 4A and 4B). These reversed pathways, which were upregulated in obesity but downregulated by Rosi, were enriched for inflammatory response processes such as the nuclear factor κB (NF-κB) pathway (Figure 4C). Interestingly, eWAT progenitor cells had downregulated pathways such as transforming growth factor β (TGF-β) signaling, conceivably reflecting a decrease in the progenitor fibroinflammatory profile (Figure 4C).^9^ In contrast, the reversed pathways that were downregulated in obesity but upregulated by Rosi were related to pathways such as PPAR signaling and adipogenesis pathways (Figure 4D). Surprisingly, we also observed an increase in ribosome and peptide chain elongation pathways in preadipocytes (Figure 4D).

Unlike eWAT, iWAT progenitor/preadipocyte cells showed a different pattern in response to Rosi (Figures S7A–S7D). In the iWAT progenitors, Rosi reversed similar numbers of up- and downregulated obesity-associated genes. However, in the iWAT preadipocytes, Rosi mainly increased the downregulated obesity-associated genes, which is opposite to the trend in eWAT preadipocytes, where Rosi primarily inhibited the upregulated genes in obesity (Figures S7A and S7B). Similar to eWAT, iWAT progenitors/preadipocytes showed a decrease in inflammation in the reserved set of genes that were downregulated by Rosi, such as activation of complement and tumor necrosis factor a (TNF-α) signaling via NF-kB (Figure S7C). Rosi-induced pathways are involved in adipogenesis and fatty acid metabolism in both iWAT APC populations (Figure S7D). In contrast to the eWAT cells, however, in iWAT we found more shared pathways between progenitors and preadipocytes in both the downregulated and the upregulated sets (Figures S7C and S7D). Interestingly, we again observed an upregulation in the ribosome, peptide chain elongation, and the eIF pathway in both iWAT progenitors and preadipocytes. Moreover, upstream regulators of translation, such as metabolism of amino acids and mTOR signaling,^36^ were also upregulated in the progenitor cells (Figure S7D).

### Rosi-dependent remodeling of the transcriptional landscape in other cell populations in the adipose stromal vascular fraction

We completed further analysis of other cell types in the adipose SVF to provide a Rosi transcriptional atlas (Figures S8 and S9). For the eWAT cells (Figure S8), we were able to assess nine populations: macrophages (LAMs, PLAMs, NPVMs, and PVMs), NKT cells, BCs, DCs, ECs, and SMCs. We found fewer than 30 genes reversed in neutrophils; therefore, we did not include the UpSet graph. Cells expressing PPARγ, such as macrophages, NKTs, ECs, and SMCs, had a robust response to Rosi^45–50^ (Figures S8A–S8I). The only exception was the BCs, which had a very small gene intersection between genes altered in obesity and those being modulated by Rosi (Figure S8F). In a manner similar to that of the eWAT progenitors/preadipocytes, the rest of the SVF had a more robust Rosi response towards the downregulation of genes previously upregulated in the ob/ob-Veh mice (Figures S8J and S8K). Collectively, the downregulated pathways involved several inflammatory and fibrosis pathways, with pathways such as IL-7 being reversed uniquely in the NKTs (Figure S8J). These results emphasized a shift towards a less inflammatory microenvironment in the eWAT after Rosi treatment. The upregulated pathways by Rosi again included the ribosome pathway, as shown in the progenitors/preadipocytes. In addition, Rosi treatment increased the expression of pathways involved in cholesterol metabolism and the respiratory electron transport chain (ETC) in macrophages (PLAMs, NPVMs, and PVM) and NKTs and also uniquely upregulated glycolysis and peroxisome pathways in PLAMs (Figure S8K).

Similar to the iWAT progenitors/preadipocytes, iWAT stromal cells had a strong upregulation by Rosi of genes downregulated in obesity, which is opposite to the eWAT cells in general (Figure S9). We were able to analyze eight populations: macrophages (LAMs, NPVMs, PVMs, and PLAMs), NKTs, BCs, DCs, and ECs. The only population we were not able to assess the Rosi response of was the neutrophils, due to a low number of genes being reversed by Rosi (Figures S9A–S9H). There were fewer pathways downregulated by Rosi; these included apoptosis, TNF-α signaling, extracellular matrix organization, and hypoxia pathways that were shared by multiple cell types, such as macrophages (NPVMs and PVMs), NKTs, and ECs (Figure S9I). The pathways upregulated by Rosi included anabolic pathways such as the ribosome, peptide chain elongation, and eIF pathway, as well as the ETC pathway, and fatty acid metabolism, across cell types (Figure S9J).

### Induction of ribosomal transcriptional signature with PPARγ agonist treatment in adipocyte progenitor cells

**Figure 5.**
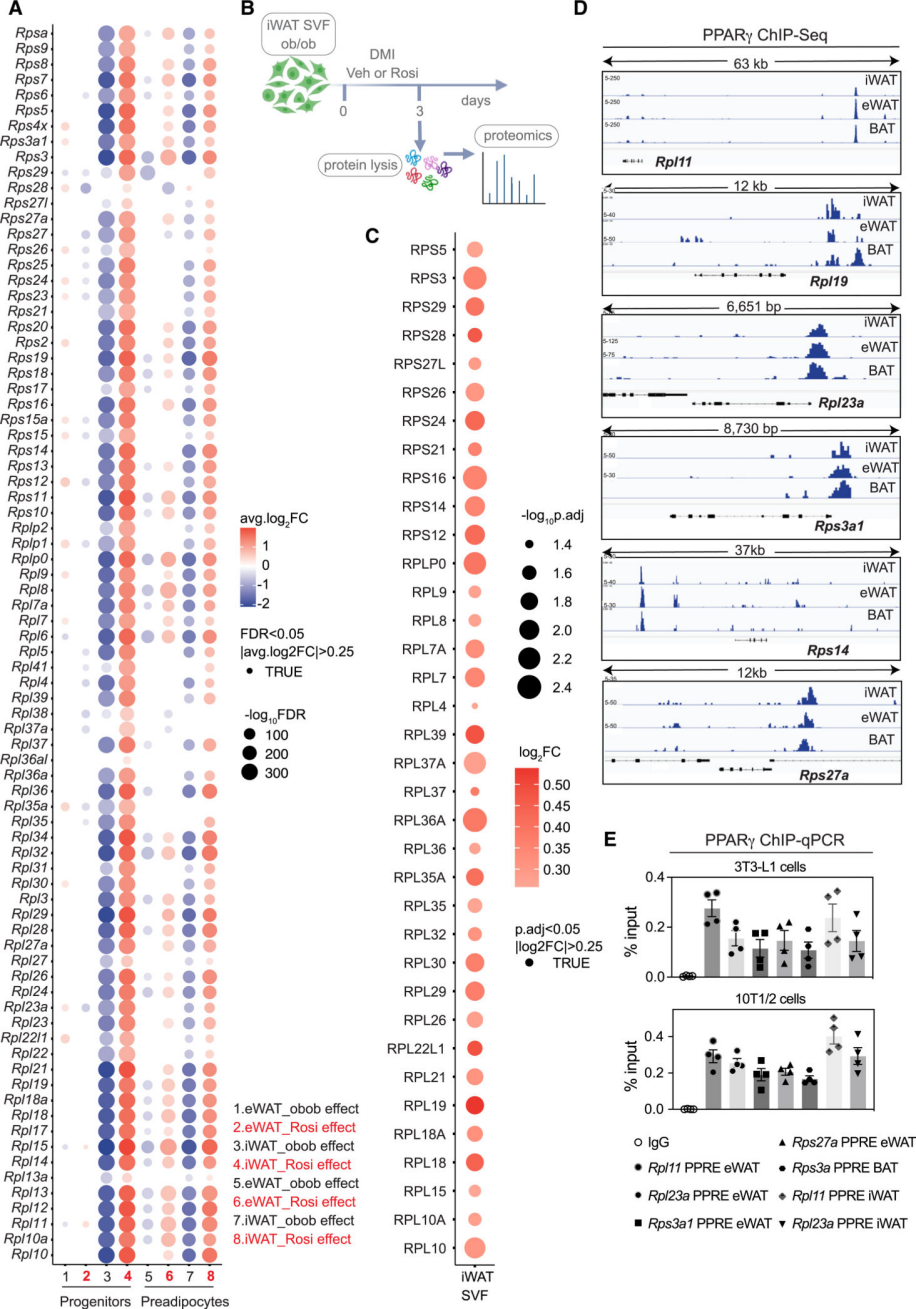
PPARγ-driven enhancement in the transcriptional network of ribosomal genes (A) Dot plot showing differentially expressed ribosomal genes influenced by the ob/ob effect or Rosi effect in eWAT and iWAT progenitors or preadipocytes all at Benjamini-Hochberg-adjusted *p* < 0.05. The size of the dots reflects the -log10(false discovery rate [FDR]) of the DEGs and the color of the dots reflects the fold change (FC) of the DEGs. (B) Schematic describing proteomics analysis of iWAT SVF isolated from ob/ob mice that were differentiated with DMI + Veh or Rosi for 3 days. (C) Dot plot of proteomic analysis showing differentially expressed ribosomal proteins regulated by Rosi in iWAT using an adjusted *p* < 0.05 and |log2FC| > 0.25. (D) PPARγ ChIP-seq showing PPARγ binding sites in close proximity to ribosomal genes: *Rpl11*, *Rpl19*, *Rpl23a*, *Rps3a1*, *Rps14*, and *Rps27a* in eWAT, iWAT, and BAT. (E) PPARγ ChIP-qPCR performed in 3T3-L1 and 10T/12 cells after 5 days of adipocyte differentiation (*n* = 4).

Given the observed potential role of PPARγ agonists in remodeling both the transcriptional landscape and the translation machinery in adipose cells, we conducted a detailed analysis of progenitor/preadipocyte cells to understand how Rosi influences the ribosomal transcriptional signature. Among the genes associated with ribosome homeostasis modulated by Rosi (Figure 5A), iWAT progenitors and preadipocyte cells from ob/ob-Rosi mice showed a significant upregulation of several small ribosomal (Rps) and large ribosomal (Rpl) transcripts (lanes 4 and 8) compared to ob/ob-Veh mice (Figure 5A). Conversely, in eWAT ob/ob-Rosi samples, there was a major upregulation of ribosome genes primarily in the preadipocyte population compared to eWAT ob/ob-Veh mice (lanes 2 and 6) (Figure 5A), likely indicating an increase in protein synthesis necessary for final adipocyte differentiation.

To test whether ribosomal proteins were induced by Rosi, we performed proteomics from the iWAT SVF of ob/ob mice differentiated in the presence or absence of Rosi (Figure 5B). We found several small and large ribosomal proteins being upregulated after Rosi treatment compared to controls (Figure 5C). To test whether ribosomal gene expression is regulated by PPARγ, we analyzed a PPARγ chromatin immunoprecipitation sequencing (ChIP-seq) dataset^51^ using differentiated SVF from BAT, eWAT, and iWAT. Reanalysis of this dataset showed a total of 41 PPARγ binding sites in the eWAT and 24 PPARγ binding sites in the iWAT that are located either at sites proximal to certain ribosome genes, such as on *Rpl23a* and *Rps3a1*, or at more distant sites of select ribosome genes such as *Rpl11*, *Rpl19*, *Rps14*, and *Rps27a* (Figure 5D). We confirmed PPARγ binding to PPAR-response elements (PPREs) on several ribosomal genes by ChIP-qPCR (Figure 5E). We also took an unbiased approach and completed motif analysis of differentially regulated ribosomal genes and found that HNF4a was highly ranked (Figure S10A), which has been previously shown to also regulate ribosomal gene expression.^52^ Although not highly ranked, PPARγ was identified through this analysis as well (Figure S10A).

### Rosi promotes translation in ob/ob mice

**Figure 6.**
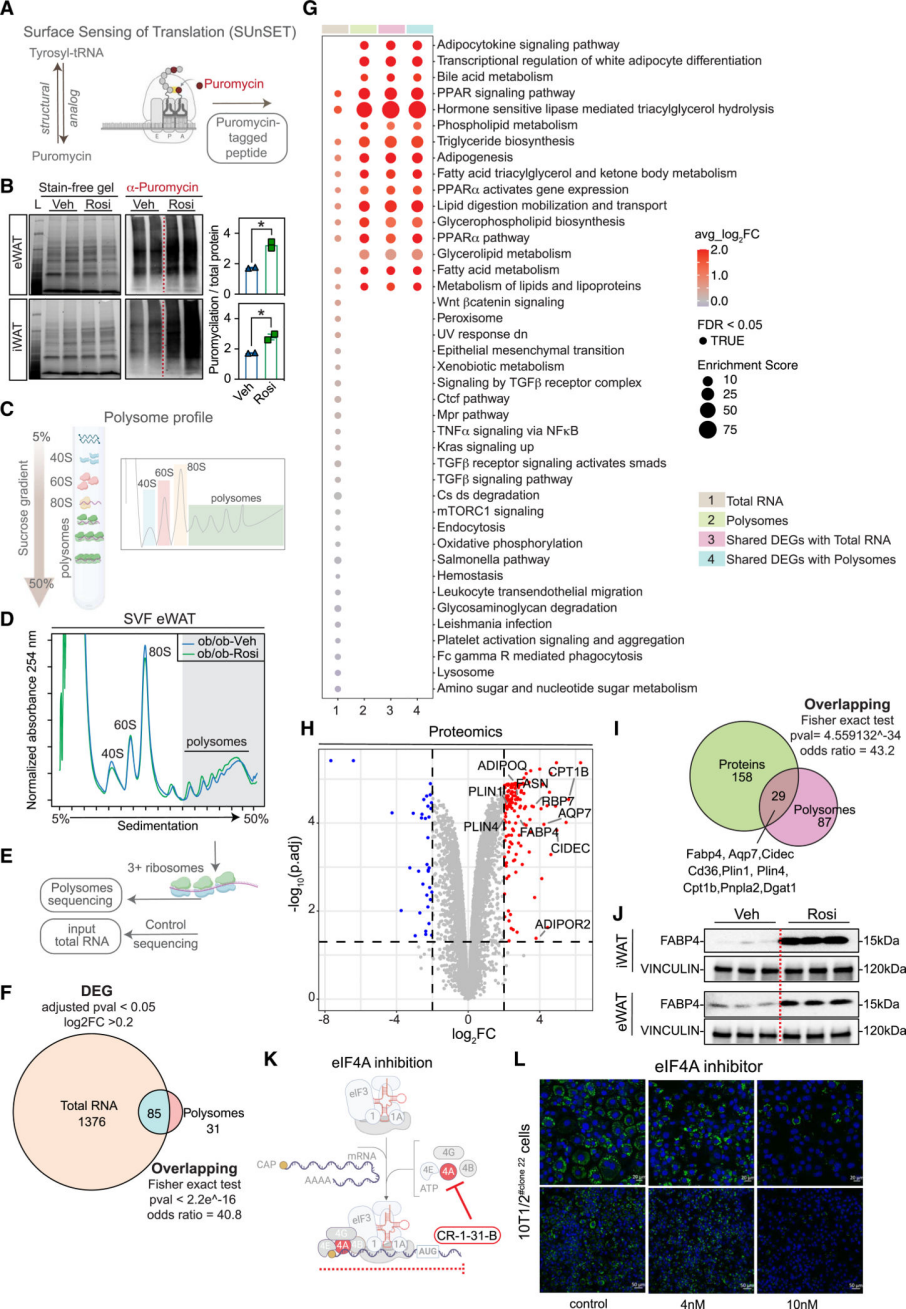
PPARγ-driven translational selectivity in Rosi treated adipocytes (A) Surface sensing of translation (SUnSET) scheme. (B) Immunoblots of eWAT and iWAT. Left: total protein in stain-free gel. Right: immunoblot anti-puromycin and puromycilation quantification. Student t test GraphPad (GP) *p* value style: *p = 0.0332. (C) Polysome profile scheme. Briefly, eWAT/iWAT SVF was differentiated for 4 days, and the cell lysate was applied to a sucrose gradient. 40S, 60S, 80S, and polysomes were separated by ultracentrifugation and fractionated. (D) Polysome profile of primary stromal vascular fraction (SVF) of eWAT after 4 days of adipocyte differentiation. (E) Experimental design for the polysome sequencing: fractions containing more than 3 ribosomes were pooled together for RNA extraction and sequencing. Control samples were input total RNA before samples were submitted to polysome fractionation. (F) Venn diagram illustrates the intersection of DEGs derived from total RNA sequencing and polysome sequencing. Both sets of DEGs satisfy the criteria of an adjusted *p* value of less than 0.05. The significance of overlap was assessed using Fisher’s exact test. (G) Dot plot visualizing the significant pathways that are enriched within the total RNA-sequencing DEG set, polysome-sequencing DEG set, and 85 shared DEG set considering all pathways at Benjamini-Hochberg-adjusted *p* < 0.05. The size of each dot corresponds to the enrichment score for each pathway, reflecting the ratio of overlapping genes to total genes within the cell-type-specific gene set, adjusted by the total number of genes detected by total RNA sequencing or polysome sequencing. Dot color represents the log2 (fold change), calculated based on the average fold change across all overlapping genes within a pathway. (H) Volcano plot of iWAT proteomics data shows differentially expressed proteins regulated by Rosi treatment at adjusted *p* < 0.05 and |log2FC| > 2. (I) Venn diagram illustrates the intersection of polysome DEGs in (F) and differentially expressed proteins in (H). The significance of overlap was assessed using Fisher’s exact test. (J) Immunoblot of FABP4 and VINCULIN from iWAT and eWAT SVF isolated from ob/ob mice and differentiated for 4 days *in vitro* with DMI plus Veh or Rosi. (K and L) Treatment of eIF4A inhibitor (CR-1-31-B) in 10T/12 clone #22 cells. Cells were differentiated with DMI + GW for 1 day and treated for 48 h with CR-1-31-B. Staining is DAPI (nuclei, blue) and LipidTox (neutral lipids, green) at low (10× bottom) and high magnifications (20× top). Confocal images: 2 wells per condition, 2 representative images per well were acquired.

Ribosomes play an essential role in translation and are dynamic and heterogenic and present a fast turnover in eukaryotes.^53^ Recent studies have shown significant heterogeneity of ribosomes,^54^ ribosome-mediated specificity,^55^ 5′ UTR functional structures directing translation,^56^ and post-translational modifications.^57,58^ To understand the role of PPARγ agonists on translation, we first measured *in vivo* protein synthesis in the adipose tissue using the surface sensing of translation (SUnSET) assay (Figure 6A). Briefly, mice received an intraperitoneal dose of puromycin—a structural analog of tyrosyl-tRNA—which is incorporated into nascent peptides that are detected by immunoblot analysis. Using this approach, we found that eWAT and iWAT showed increased puromycin labeling, indicating an increase in protein synthesis in the ob/ob-Rosi-treated mice compared to ob/ob-Veh controls (Figure 6B).

To determine whether remodeling in the ribosome-related pathways had an impact on translation and to understand which mRNA networks were differentially translated in response to Rosi, we carried out polysome profiling followed by RNA-seq (Figure 6C). Briefly, cells from SVF of ob/ob mice were differentiated with either DMI-Veh or DMI-Rosi for 4 days *in vitro* (to mimic the *in vivo* Rosi treatment). Polysome profiles from eWAT SVF of ob/ob-Veh and ob/ob-Rosi were found to be similar in both eWAT (Figure 6D) and iWAT (Figure S10B). Subsequently, we conducted polysome sequencing on eWAT SVF to profile highly translating mRNAs. This choice was driven by eWAT’s (1) robust tissue environment remodeling after Rosi treatment (Figures 1G and 2B) and (2) substantial difference in *ex vivo* adipogenesis between Veh and Rosi coupled with (3) its comparatively less understood response to Rosi relative to iWAT. Briefly, input total RNA (control samples) and mRNA from polysome fractions containing more than three ribosomes were extracted and sequenced (Figure 6E). We completed differential gene expression analysis from the polysome fractions between the ob/ob-Rosi group and the ob/ob-Veh group. As a comparison, we also measured differential gene expression on total RNA. In both datasets, transcripts with an adjusted *p* value below 0.05 and a log2 fold change (FC) exceeding 0.2 were designated as differentially expressed. We found >1,400 differentially expressed genes (DEGs) with total RNA and 116 DEGs in the polysome fraction, suggesting translational buffering (Table S1). Translational buffering opposes the impact of alterations in mRNA levels on the proteome by compensating, equilibrating, and offsetting translation^59^ (Figure 6F). These findings suggest significant selectivity in which mRNAs are found in the polysome fraction and are highly translated in response to Rosi.

Among the pathways enriched in the DEGs identified from total RNA-seq (input) are lysosomes, immune response (TNF-α and TGF-β), and oxidative phosphorylation pathways (Figure 6G). In contrast, DEGs being highly translated in the polysome fraction were enriched for more selective pathways pertaining to adipocyte functions, including adipokine signaling pathway, triglyceride biosynthesis, and adipogenesis (Figure 6G). The 85 shared DEGs were enriched for the same pathways as the ones enriched by polysome fraction DEGs, including glycerophospholipid biosynthesis (pathway-wise mean log2(FC) of 1.21 with total RNA FC values and 1.35 with polysome fraction FC values) and PPARa pathway (mean log2FC of 1.54 with total RNA FC values and 2.0 with polysome fraction FC values) (Figure 6G). These results highlight how eWAT switches its translatome from inflammatory pathways to a metabolic/adipogenic profile after Rosi treatment.

To validate the above findings from eWAT SVF, we also examined the response in the primary SVF cells in both eWAT and iWAT by qPCR (Figures S10C–S10E). We selected the highly ranked targets based on the degree of change between groups (log2FC), which included several fat-specific genes such as *Adipoq*, *Arxes1*, *Fabp4*, *Plin1*, and *Aqp7*. Surprisingly, we found unique patterns of selectivity in the eWAT SVF, with *Adipoq*, *Arxes1*, and *Fabp4* presenting higher expression in the polysome fraction than in the total RNA, highlighting robust translational induction, whereas *Plin1* and *Aqp7* showing a more modest trend (Figure S10D). In contrast, the iWAT SVF presented a similar pattern of higher polysome occupancy for all those targets, emphasizing a fat-depot specialization in response to Rosi (Figure S10E) and potentially explaining its stronger response to Rosi. We also ranked by fold change the transcripts present only in the polysomes (Figure S10F), highlighting, for instance, the presence of important adipose genes such as *Clstn3*.^60^

Next, we addressed whether changes in translation lead to similar changes at the protein level by completing a proteomics analysis (Figure 5B). We found that several adipogenic proteins were upregulated by Rosi (Figure 6H), confirming a positive correlation between translational activity (Figures S10D and S10E) and protein expression and showing a fine regulation of selected proteins being upregulated between the polysome and the proteomics (Figure 6I). Since most of these targets are direct targets of PPARγ, we were also able to validate the expression by immunoblot of FABP4 (Figure 6J).

Our observation that ob/ob mice have reduced expression of ribosomal factors as measured by scRNA-seq compared to lean mice suggests that inhibition of translation may be a driving factor that leads to impaired adipogenesis in obesity. To test this hypothesis, we incubated 10T1/2^clone#22^ cells with a translation inhibitor targeting eIF4A (CR-1–31-B) as described in the STAR Methods (Figures 6K and 6L). At day 3 of differentiation, control cells showed significant differentiation, while cells treated with 10 nM CR-1–31-B had few lipid droplets (Figures 6K and 6L). Notably, at 25 nM, we noted cell death after 48 h (data not shown) (Figure S10G). Using a similar paradigm, we repeated the experiment with sorted iWAT DPP4^+^ and DPP4^-^ cells and found that control (DPP4^+^ and DPP4^-^) cells from ob/ob-Rosi mice had increased adipogenic potential compared to ob/ob-Veh mice (Figure S10H). Interestingly, after translation was blocked, both ob/ob-Veh and ob/ob-Rosi cells showed similar differentiation (Figure S10H). We noted that CR-1–31-B had a stronger effect on differentiation in 10T1/2 cells, perhaps because primary cells were strongly committed to the adipogenic lineage, showing lipid droplets within 24 h; therefore, blocking translation did not impact the existing lipid droplets.

### Identification of GC-rich motifs in the 5′ UTR of mRNAs with enhanced translational efficiency induced by Rosi

**Figure 7.**
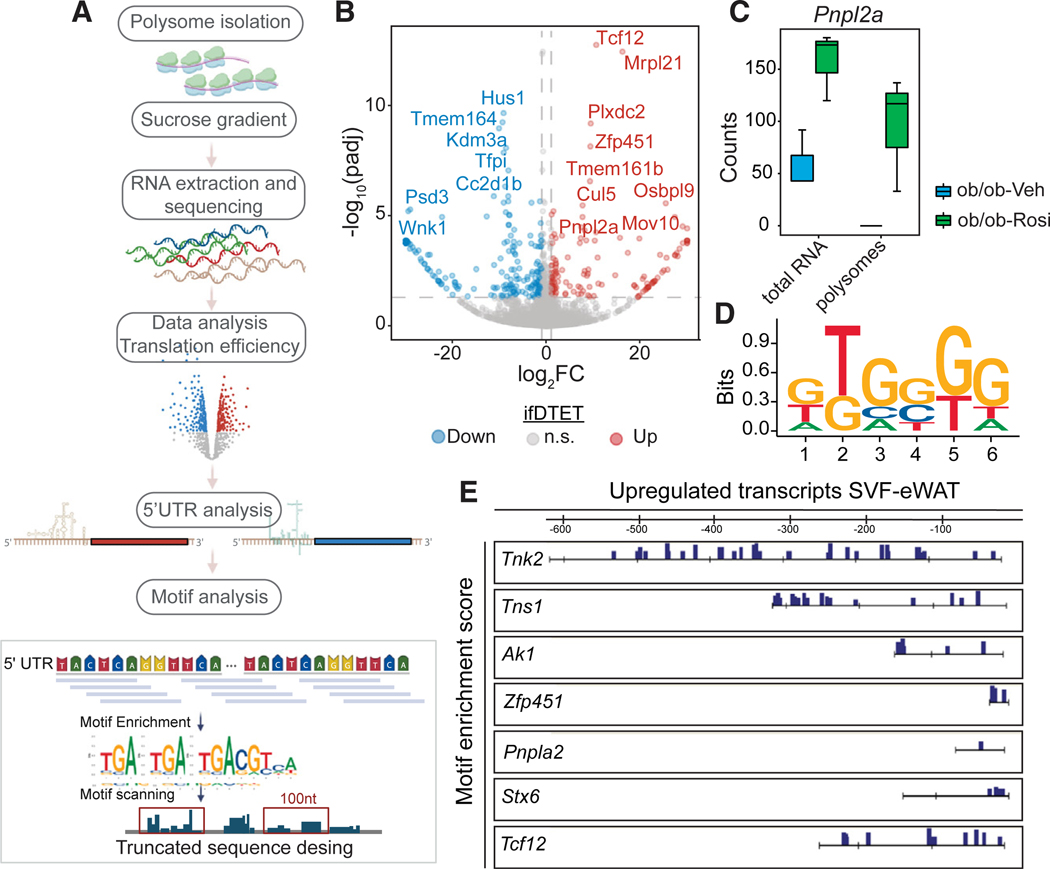
Rosi-induced translation efficiency and identification of G-rich motifs in the 5′ UTR (A) Schematic overview of polysome profiling and sequence feature analysis in the 5′ UTR under acute Rosi treatment. (B) Volcano plot illustrating the differentially translated transcripts between Rosi and Veh treatment. The x axis shows the log2 transformed fold change, while the y axis shows the adjusted *p* value. The red and blue dots label the up- and downregulated transcripts, respectively. (C) Boxplot shows the normalized read count on the *Pnpla2* transcript between different treatments in total RNA and polysome fractions. (D) Sequence motif enriched in the 5′ UTR of upregulated transcripts. (E) The feature map of the 5′ UTR of selected upregulated transcripts. Each deep blue bar indicates a significant site with a *p* value lower than 0.05 calculated by the scan-matrix program.

Translation is a predominant process for protein synthesis that regulates the global level of gene expression products.^61^ To determine whether Rosi impacts translation efficiency, we reanalyzed the polysome sequencing data by normalizing polysome-enriched RNAs to total RNA. This approach allowed us to systematically identify transcripts with differential translation efficiency (DTETs) (Figure 7A, see STAR Methods^62^). A total of 135 transcripts had increased translational efficiency by Rosi and 206 were downregulated (Figure 7B). Intriguingly, upregulated transcripts were not necessarily direct targets of PPARγ (Figure 7B), highlighting an enhancement in translation efficiency independent of the increase in gene expression caused by the PPARγ-agonist treatment. Notably, one of the isoforms of *Pnpla2* (ENSMUST00000169665) was identified as a DTET after Rosi treatment (Figure 7C). *Pnpla2* encodes adipose triglyceride lipase (ATGL), a pivotal enzyme in triglyceride hydrolysis.^63^ Translation initiation is controlled by primary sequences or secondary structures in the 5′ UTR mRNAs.^56^ Analysis of the 5′ UTR showed an enrichment of hexamers as measured by k-mer enrichment analysis (Figure 7A). Interestingly, we found that the 5′ UTR of Rosi-upregulated DTETs contains guanine (G)-rich sequences (Figure 7D), which are capable of forming G quadruplexes (G4) and other secondary structures that serve as roadblocks in the translation process.^64^ Secondary structures such as G4 can impact the ability of the initiation machinery to scan the UTR to identify the start codon.^65^ For instance, DNA G4 structures are lost during stem cell differentiation to drive cellular specialization by enhancing translation.^66^

To verify whether the enrichment scores were robust when choosing different background sets, we calculated the correlations of the hexamers’ enrichment scores and observed strong correlations (Figure S11A). In addition, we performed the same analysis in coding regions (CDS) and 3′ UTRs (Figure S11A). The enriched hexamers in the CDS of Rosi-induced DTETs showed a strong AG-rich motif, whereas the motif in the 3′ UTR was U rich (Figures S11B and S11C), highlighting the diverse translation regulation in different RNA regions. We then focused on the 5′ UTR because it is well established that secondary structures in mRNAs are essential to regulate translation initiation.^67^ We decided to characterize the transcripts containing this G-rich motif in the upregulated targets (Figure 7A; see STAR Methods). We identified several transcripts presenting the G-rich motif in the 5′ UTR (Figure S11D). Remarkably, we found several zinc fingers containing transcripts, such as *Zfp451*, *Zfp292*, *Zfp932*, *Zfp825*, *Zfp131*, *Zfp654*, and *Zfp516* (Figures 7E and S11D). *Zfp451* and *Zfp516* in particular are directly involved in driving stem cell differentiation and activation of UCP1 expression, respectively.^68,69^ In addition, *Syntaxin 6* (*Stx6*), *Tensin 1* (*Tns1*), *Periostin* (*Post*), and *Pnpla2* (coding for ATGL) were directly involved in adipose-specific processes such as Glut4 trafficking,^70^ cell-cell interactions,^71^ and lipid metabolism.^63,72^

## DISCUSSION

Adipose tissue dysfunction represents a distinct risk factor for the development of type 2 diabetes. In particular, adipocyte hypertrophy plays a pivotal role in disrupting metabolic adaptability. While adipose tissue has the capacity to expand, there is a finite threshold in its capacity to absorb excess lipids, contributing to the onset of metabolic syndrome.^73–76^ The use of TZDs as an antidiabetic treatment stimulates the production of new adipocytes, diminishes inflammation, and enhances metabolic adaptability. Notably, Rosi stands out as a potent agent that bolsters insulin sensitivity in peripheral tissues, including both muscle and adipose tissue.^19^ Our acute treatment strategy highlights the strong therapeutic effects of Rosi in normalizing glucose metabolism and that these changes coincide with the induction of ribosomal factors in adipose tissue. Our observation that transcriptional expression of ribosomal factors is reduced in adipocyte progenitors and preadipocytes of obese mice may explain why obese mice develop adipocyte hypertrophy, as we have now shown direct evidence that inhibition of translation blocks adipocyte differentiation. Thus, one could speculate that reduced expression of ribosomal factors could prevent lipid storage in emerging adipocytes, leading to accumulation of energy in existing adipocytes that develop hypertrophy.

Our single-cell analysis shows how cells from the SVF respond to Rosi during obesity in the adipose tissue. We found extensive remodeling of many cell types in the SVF of both eWAT and iWAT, including a reduction in lipid-associated macrophages/IMs after Rosi treatment in obese mice. The decrease of inflammatory cells is a remarkable function of Rosi, and it is essential to restore the immune tonus in the adipose tissue. Notably, our findings highlight a role for Rosi in enhancing ribosomal gene expression and subsequent translational efficiency. It is noteworthy that of the 1,461 differentially expressed transcripts that respond to Rosi, only 85 are enriched in the polysome fraction, suggesting selectivity of mRNAs that are being highly translated. Moreover, these mRNAs are enriched in pathways involved in adipogenesis, lipid metabolism, hormone-sensitive lipase and triglyceride hydrolysis, adipocytokine signaling, and bile acid metabolism. The drivers of cell-type-selective translational selectivity are not well documented but are of significant interest in their potential for providing therapeutic targets for metabolic diseases. The specialization of ribosomes is a current focal point across various fields of study.^53^ Therefore, further studies to understand how ribosomes specific to fat tissue select the mRNA networks for translation are needed.

How post-transcriptional processes control the precise balance of protein production within adipocytes is not well understood. Our analysis suggests that, while there is induction of translation, there is significant translational buffering. This buffering prevents abrupt fluctuations in protein levels due to changes in gene expression, ensuring that translation is fine-tuned and responsive to specific cellular conditions. Translation buffering can be achieved through factors like ribosome pausing,^77^ increasing mRNA stability,^78^ or regulating the availability of transfer RNA (tRNA) molecules.^79^ Our data suggest that Rosi-induced transcripts undergo translational buffering, where there is a reduction in translation rate compared to the total mRNA (Table S1). Surprisingly, although a large number of genes are transcriptionally altered by Rosi, we found that in the eWAT there is a decrease in the translation of several immune-related pathways, and instead, the polysomes are enriched with adipogenic-related targets allowing these genes to be selectively translated (Figure 6). Ribosomes somehow bypass the overwhelming transcriptional remodeling of Rosi and selectively induce the translation of essential adipose-specific targets that promote fat homeostasis.

The regulation of translation specific to the adipose tissue is an area that lacks mechanistic understanding. For example, in 3T3-L1 cells during adipogenesis, enhancement of the ribosome machinery and a dynamic regulation of polysomes versus free mRNA fraction seem essential to drive full adipocyte differentiation.^80,81^ In addition, initiation factors, such as eIF6, control the translation of transcriptional factors C/EBPβ and C/EBPδ in a G/C-rich 5′ UTR manner, contributing to lipid metabolism homeostasis.^38^ Our studies propose that Rosi regulates fat-specific translation through a direct mechanism where PPARγ drives the expression of ribosomal transcripts and builds up the machinery necessary to translate specialized fat mRNAs. In conclusion, we propose an exciting role for TZDs (Rosi) in regulating the translation machinery in the adipose SVF. Our studies suggest that Rosi not only drives the fat-transcription networks but also promotes translation in a very precise and sophisticated way to ensure fat cell specialization. This opens up a new perspective on adipose tissue translation homeostasis and its adaptability to diverse stressors.

### Limitations of the study

Our finding that obese mice have reduced expression of ribosomal factors compared to lean mice in progenitors and preadipocytes was remarkable. Further analysis to address whether obesity decreases protein expression by western blot analysis or mass spectrometry is needed to further validate these findings. Furthermore, polysome profiling of lean and obese mice is needed to provide a better understanding of how translation is impacted. In addition to that, even though we showed increase at protein levels of ribosomal proteins after Rosi treatment compared to ob/ob-Veh, we did not find a significant increase in the peaks of monosomes or polysomes in the polysome profile. This finding could imply that either ribosomal proteins are not being incorporated into ribosomes or there is an increased turnover of ribosomes in ob/ob-Rosi mice compared to ob/ob-Veh mice. Finally, our analysis of the 5′ UTR mRNAs showed structures that could modulate the rate of translation, such as the G quadruplexes. These structures normally are involved as repressors of translation; however, we found an upregulation in translation efficiency. This could highlight a yet undescribed role of Rosi driving the expression of proteins that may be able to reverse these structures.

## STAR★METHODS

### RESOURCE AVAILABILITY

#### Lead contact

Requests for further information and resources or reagents should be directed to the lead contact, Claudio Villanueva, at cvillanueva@ucla.edu.

#### Materials availability

This study did not generate new unique reagents.

#### Data and code availability

- scRNA-seq and polysome RNA-seq data have been deposited at GEO: GSE267192 and GSE268057 and are publicly available as of the date of publication.
- All original code has been deposited at Zenodo at https://doi.org/10.5281/zenodo.13926724 and is publicly available as of the date of publication.
- Any additional information required to reanalyze the data reported in this paper is available from the lead contact upon request.

### EXPERIMENTAL MODEL AND STUDY PARTICIPANT DETAILS

#### Cells

3T3L1 cell line was obtained from ATCC (CL-173). C3H/10T1/2 cell line was obtained from ATCC (Clone 8, CL-226). 10T1/2 cells were subjected to limiting dilution to identify a monoclonal cell population exhibiting high adipogenic potential (based on lipid droplet formation and PPARγ target gene expression). 10T1/2 clone#22 cells were gifted from Tontonoz Lab.

#### Mice

Adult C57BL/6J (12-week-old) male mice (stock #000664) and *Lep*^*ob*^*/Lep*^*ob*^ (stock #000632) were acquired through Jackson Laboratories. All mice were housed at a maximum of 5 animals per cage in temperature-controlled rooms under a 12-hour light/dark cycle and provided water and chow *ad libitum.* All mouse procedures were performed under animal study proposals approved by the University of California, Los Angeles Animal Research Committee (ARC 2019–066).

### METHOD DETAILS

#### Rosiglitazone treatment

To examine the effects of PPARγ agonist, C56BL/6 and *Lep*^*ob*^*/Lep*^*ob*^ mice were given 30 mg/kg rosiglitazone (Sigma R2408), or vehicle [2.6% methylcellulose (StemCell Technologies, M3120) diluted 1:5 in Dulbecco’s modified medium (GIBCO)] by oral gavage in the morning and evening for three consecutive days as previously described.^88^

#### Stromal vascular fraction isolation for scRNASeq

To ensure high quality and viability of the single cell suspensions, stromal vascular fractions were isolated as previously described^89^ with small modifications. Briefly, 300mg of the white adipose tissue (eWAT or iWAT) were collected (small biopsies from several locations of the fat depot to represent tissue environment), cut into small pieces, suspended in phosphate-buffered saline (PBS) (calcium, magnesium free) (GIBCO) containing 2mg/mL of type II collagenase (Worthington), and digested for 40 minutes at 37°C with agitation (100 rpm). The resulting dissociated tissue was resuspended in DMEM (GIBCO) with 10% FBS, gently mixed, passed through 100μm strainers, and centrifuged at 150g for 8 min at 4C. Next, supernatant and floating adipocytes were removed, and pellet was resuspended in DMEM (GIBCO) with 10% FBS and centrifuged at 150g for 8 min at 4C. Red blood cells were lysed using ACK lysis buffer (GIBCO). Lysate pellets were first washed with DMEM (GIBCO) with 10% FBS, centrifuged at 150g for 8 min at 4C and then PBS with 0.04% BSA (Gemini). The resulting pellets were finally resuspended in 200uL of PBS, 0.04% BSA (Gemini), passed through 40μm tip-strainers, counted, and further used for 10X 3’GEX library preparation and sequencing.

#### 10X 3’GEX library preparation and sequencing

The Chromium Single Cell Gene Expression Solution upgrades short read sequencers to deliver a scalable microfluidic platform for 3ʹ digital gene expression by profiling 500–10,000 individual cells per sample. A pool of ~3,500,000 10x Barcodes are sampled separately to index each cell’s transcriptome. It is done by partitioning thousands of cells into nanoliter-scale Gel Beads-in-emulsion (GEMs), where all generated cDNA shares a common 10x Barcode. Libraries are generated and sequenced from the cDNA and 10x Barcodes are used to associate individual reads back to the individual partitions. In addition to the poly(dT) primer that enables the production of barcoded, full-length cDNA from poly-adenylated mRNA, the Single Cell 3ʹ v3.1 Gel Beads also include two additional primer sequences (Capture Sequence 1 and Capture Sequence 2), that enable capture and priming of Feature Barcoding technology compatible targets or analytes of interest. Only the poly(dT) primers are used in this protocol for generating Single Cell 3ʹ Gene Expression libraries. GEMs are generated by combining barcoded Single Cell 3ʹ v3.1 Gel Beads, a Master Mix containing cells, and Partitioning Oil onto Chromium Next GEM Chip G. To achieve single-cell resolution, cells are delivered at a limiting dilution, such that the majority (~90–99%) of generated GEMs contain no cell, while the remainder largely contains a single cell. Immediately following GEM generation, the Gel Bead is dissolved, primers are released, and any co-partitioned cell is lysed. Primer containing (1) an Illumina TruSeq Read 1 (read 1 sequencing primer), (2) 10x Barcode, (3) 12nt unique molecular identifier (UMI), and (3) 30nt poly(dT) sequence are mixed with the cell lysate and a Master Mix containing reverse transcription (RT) reagents. Incubation of the GEMs produces barcoded, full-length cDNA from poly-adenylated mRNA. After incubation, GEMs are broken, and pooled fractions are recovered. Silane magnetic beads are used to purify the first-strand cDNA from the post GEM-RT reaction mixture, which includes leftover biochemical reagents and primers. Barcoded, full-length cDNA is amplified via PCR to generate sufficient mass for library construction. Enzymatic fragmentation and size selection are used to optimize the cDNA amplicon size. TruSeq Read 1 (read 1 primer sequence) is added to the molecules during GEM incubation. P5, P7, a sample index, and TruSeq Read 2 (read 2 primer sequence) are added via End Repair, A-tailing, Adaptor Ligation, and PCR. The final libraries contain the P5 and P7 primers used in Illumina bridge amplification. A Chromium Single Cell 3’ Gene Expression library comprises standard Illumina paired-end constructs which begin and end with P5 and P7. The 10x Barcode and 12 bp UMI are encoded in Read 1, while Read 2 is used to sequence the cDNA fragment. Sample index sequences are incorporated as the sample index read. TruSeq Read 1 and TruSeq Read 2 are standard Illumina sequencing primer sites used in paired-end sequencing. These libraries were sequenced using Illumina’s NovaSeq6000 platform in paired end 2 × 100bp configuration. Data quality check was done on Illumina SAV, and data de-multiplexing was performed with Illumina Bcl2fastq v2.19.1.403 software.

#### scRNA-seq data processing and quality control

The 10X Genomics Cell Ranger version 3.0.2 single-cell software^87^ was used to perform sample demultiplexing sequencing alignment to mouse genome mm10, filtering, and unique molecular identifier (UMI) counting to generate gene count matrices. Single cells were identified from background noise by filtering on the proportion of mitochondrial reads (threshold: < 25%), the number of UMI (thresholds: 700–22,000), and the number of detected genes (thresholds: 200–6,000).

#### Cell clustering and cell type identification

The cell clustering and cell type identification were performed using the Seurat R package version 4.0.2. The Louvain algorithm was employed to determine cell clusters based on similarities in transcriptome patterns, and the resulting clusters were visualized using t-Distributed Stochastic Neighbor Embedding (t-SNE) and Uniform Manifold Approximation and Projection (UMAP). Highly variable genes selected using the FindVariableFeatures function with default parameters were subjected to principal component analysis (PCA). The number of principal components used for Louvain clustering and subsequent visualization was determined with the Jackstraw permutation approach (*n*=25 for clustering of all cells and *n*=15 for APC sub-clustering). Since all samples were processed in the same batch and sequenced together, and we did not observe any disagreement in cell type alignment among the replicates in each group and between the samples in two (WT-Veh vs ob/ob-Rosi) of the three experimental groups. Therefore, there is a lack of evidence for batch or technical effect in our dataset to justify data integration. The only difference we observed was between the ob/ob-Veh group against the other two groups, which represents biological effects that should not be removed. For this reason, we did not use integration across groups in order to preserve the biological effect. Cell type identities of the clusters were resolved by comparing the cell cluster-specific marker genes expressed in each cluster in our own dataset, identified with a Wilcoxon rank sum test, with known cell-type-specific markers curated from literature, single-cell atlases, and previous studies in the white adipose tissue. For a gene to be considered in the cell cluster marker analysis, it had to be expressed in at least 10% of the single cells from the cluster of interest and exhibit at least a 0.25 log-fold change in the cell cluster of interest compared to other cells. Multiple testing was corrected using the Benjamini-Hochberg method to estimate the false discovery rate (FDR).

#### Identification of differentially expressed genes (DEGs), pathways, and enriched motifs

To determine which genes were affected by genetic background or rosiglitazone treatment, we compared the cell transcriptome of each cell type between age groups using a Wilcoxon rank sum test. To be considered in the analysis, a gene had to be expressed in at least 10% of the single cells from at least one of the two groups for that cell type and there had to be at least a 1.1-fold change in gene expression between the groups. Multiple testing correction was done using the Benjamini–Hochberg method to estimate FDR. To assess pathway enrichment, we performed Fisher’s exact test to determine the overlap between the DEGs and pathways from KEGG, REACTOME, BIOCARTA, and HALLMARK. Multiple testing correction was performed using the Benjamini-Hochberg method to estimate FDR. The enrichment score was calculated as the number of overlapped genes divided by the number of genes in our cell type-specific gene set, multiplied by 20,000, and then divided by the total number of genes in the pathway. For the Upset plot illustrating the intersection of differentially expressed genes (DEGs), all graphs are at Benjamini-Hochberg-adjusted *p*-value < 0.05. The 4 categories include upregulated DEGs in obese mice compared to lean mice (ob/ob_UP), downregulated DEGs in obese mice compared to lean mice (ob/ob_DOWN), upregulated DEGs in response to Rosi treatment compared to ob/ob-Veh (Rosi_UP), and downregulated DEGs in response to Rosi treatment compared to ob/ob-Veh (Rosi_DOWN) (see STAR Methods). Horizontal bars (set size) indicate total DEGs for each cluster in each plot. In the UpSet plots, dots point to the specific clusters for which the vertical bars for DEG counts are shown, and vertical lines between dots represent the intersections between two or more clusters. The blue circle signifies the set of DEGs that are upregulated in obese mice and downregulated in response to rosiglitazone treatment. The red circle represents the DEGs that are downregulated in obese mice and upregulated following rosiglitazone treatment. For the reversed pathways (upregulated or downregulated by Rosi), the size of each dot corresponds to the enrichment score for each pathway, reflecting the ratio of overlapping genes to total genes within the cell type-specific gene set, adjusted by a scale factor of 20,000, and then divided by the total number of genes within the pathway. The color of the left side of each dot represents the log2(fold-change), calculated based on the average fold change across all overlapping DEGs within a pathway in obese mice compared to lean mice. Color of the right side of each dot represents the log2 (fold-change), calculated based on the average fold change across all overlapping DEGs within a pathway in response to rosiglitazone treatment. To identify motif sequences enriched in the promoter regions of the differentially expressed ribosomal genes that are influenced by the ob/ob effect or Rosi effect in eWAT and iWAT progenitors or preadipocytes, we applied findMotifs.pl function of HOMER^83^ with default and modified parameters (promoter region used for motif finding: −2000 and 2000; motif length: 12,13,14) to explore *de novo* and known motif sequences.

#### Regular stromal vascular fraction and mature adipocyte isolation

Stromal vascular fractions were isolated as previously described.^90^ Briefly, entire WAT depots were cut into small pieces, suspended in DMEM (GIBCO) + 50 mM HEPES (GIBCO) + 1mg/mL type II collagenase (Sigma), + 1% BSA - low fatty acid (Gemini), and digested for 30 minutes at 37°C with agitation (120 rpm). The resulting dissociated tissue was passed through 100mm strainers, and adipocytes were either collected and frozen in Trizol (ThermoFisher) for RNA extraction or removed from the supernatant by centrifugation (750g, 10min, 4C). After a second filtration with 40μm strainers, red blood cells were lysed using ACK lysis buffer (GIBCO). The resulting pellets were processed further for flow cytometry, sorting or primary cell culture.

#### Flow cytometry and sorting

Cells were analyzed for cell-surface markers using fluorophore-conjugated antibodies (BioLegend, eBioscience). Cell surface staining was performed in HBSS (GIBCO). Flow cytometry was performed using the Attune NxT and data were analyzed with FlowJo software (BD). Cell surface and intracellular staining were performed using the following fluorophore-conjugated antibodies: (a) macrophages panel^8^: CD45.2 (104), TCRb (H57–597), CD3 (17A2), CD19 (6D5), NK1.1 (PK136), Ly6G (1A8), CD11c (N418), CD11b (M1/70), CD88 (20/70), CD9 (MZ3), Tim-4 (RMT4–54). (b) progenitors: Lineage negative: CD45.2 [104], and CD31 [390], PDGFRα (APA5), DPP4 (H194–112), ICAM-1 (YN1/1.7.4), CD36 (HM36). For sorting, cell suspensions were stained with: CD45.2 [104], and CD31 [390], PDGFRα (APA5), DPP4 (H194–112), and DAPI (AAT Bioquest) and sorted on FACSAria III (BD).

#### Confocal

Sorted cells were plated in a 96-well plate and culture in DMEM (GIBCO), 5μg/mL insulin (GIBCO), 10% FBS (Omega FB#11), 1% PenStrep (GIBCO), and 50μg/mL Primocin (Invivogen) until confluency. Specific differentiation cocktails included: (a) complete adipogenic cocktail (0.5mM 3-isobutyl-1-methylxanthine (Sigma), 1mM dexamethasone (Sigma), 5μg/mL insulin (GIBCO), or (b) DI (dexamethasone (Sigma), and 5μg/mL insulin (GIBCO), or (c) minimal media (5μg/mL insulin (GIBCO). Differentiation media was added for two days and then replaced with minimal media (DMEM, 10% FBS, 5μg/mL insulin) until day 4 of differentiation. Cells were fixed with 4% paraformaldehyde, permeabilized with 0.1% Triton, and stained with LipidTOX Green Neutral Lipid (Invitrogen) and DAPI (AAT Bioquest). For macrophage staining, cells from the adipose tissue were dissociated as previously described. Upper floating layer containing mature adipocytes was collected and plated in an 8-well chamber (Nunc Lab-Tek, Sigma) previously treated with poly-L-lysine. After overnight incubation at 37C, slides were washed four times with phosphate-buffered saline (PBS), fixed with 4% paraformaldehyde, permeabilized with 0.1% Triton, blocked with normal donkey serum 5% (Jackson Immuno Research) and stained overnight with PLIN1 (rabbit, 3526, Abcam), and F4/80 (rat, BM8, BioLegend). Next, slides were washed four times with PBS and incubated for one hour with LipidTOX Green Neutral Lipid (Invitrogen), DAPI (AAT Bioquest), donkey anti-rat IgG Alexa Fluor 568 (Abcam), and donkey anti-rabbit IgG Alexa Fluor 647 (Abcam). Images were acquired using Zeiss LSM900 microscope.

#### Histology and lipid droplet quantification

Tissues were fixed for 48h in 10% buffered formalin (ThermoFisher), after which they were washed with 70% ethanol (ThermoFisher), sectioned in paraffin (10 μm thickness for adipose tissues and 5 μm for liver), and stained with hematoxylin and eosin (H&E). The lipid droplet cell area from livers and progenitors/preadipocytes from confocal images were quantified using ImageJ.

#### Glucose tolerance test

For glucose tolerance tests, mice were fasted for 6 hours prior to the challenge with glucose (1 g/Kg mouse) via intraperitoneal injection. Blood glucose levels were assessed by tail vein bleeding using a glucometer (Accu-Chek). AUC was determined using Prism software (GraphPad).

#### μPET/μCT

*Lep*^*ob*^*/Lep*^*ob*^ mice (stock #000632) (12 weeks old, male) treated with either vehicle (*n*=6) or rosiglitazone (*n*=6) were fasted for six hours, prior to intravenous injections via tail vein with 85–90 μCi of [^18^F]-FDG that had been mixed with glucose based on the weight of the mouse (1g/Kg). Following a 50-minute conscious uptake of [^18^F]-FDG, mice were anesthetized with 2% vaporized isoflurane, and PET (energy window 350–650 keV, 10-min static scan) and CT (voltage 80 kVp, current 150 μA, 720 projections, 200μm resolution, scan time 1 min) images were acquired on a GNEXT PET/CT scanner (Sofie Biosciences, Dulles, VA). The PET images were reconstructed using a 3D-Ordered Subset Expectation Maximization (OSEM) algorithm (24 subsets and 3 iterations), with random, attenuation, and decay correction. The CT images were reconstructed using a modified Feldkamp algorithm. Amide software was used to analyze co-registered uPET/uCT images, and a full body panel was generated by placing ROIs for the brain, blood, liver, left and right kidney, bladder, muscle, left and right lung, gastrointestinal tract, and adipose tissues. Visual representation of μPET signal from adipose tissues was generated using ORS Dragonfly software (Object Research Systems Inc, Montreal, Canada).

#### Surface Sensing of Translation (SUnSET)

The assay was performed as previously described with small modifications.^91^ Briefly, mice were intraperitoneally injected with puromycin (40 nmol/g) (Santa Cruz) and returned to their cages with food/water *ad libitum* for one hour. Next, brown, epididymal, and inguinal adipose tissue were collected, and proteins were lysed using radioimmunoprecipitation assay buffer (RIPA) (50mM Tris-HCl pH 7.6, 150mM NaCl, 0.1% sodium dodecyl sulfate, 0.5% sodium deoxycholate, 1% NP40 and freshly added halt protease/phosphate inhibitor cocktail (ThermoFisher). Immunoblots were performed using 20μg of protein and total protein was assessed using UV light exposure to Mini-Protean TGX Stain-free gels (Biorad). Puromycilation was detected using primary anti-puromycin antibody (12D10, Sigma), and HRP AffiniPure Goat anti-mouse IgG, Fcg subclass 2a specific (Jackson Immuno Research). Quantification was performed using ImageJ.

#### Immunoblot

Stromal vascular fraction of *Lep*^*ob*^*/Lep*^*ob* was^ mice was isolated, and primary cells were grown to confluency. Cells were then treated to differentiate with complete adipogenic cocktail (0.5mM 3-isobutyl-1-methylxanthine (Sigma), 1μM dexamethasone (Sigma), 5μg/mL insulin (GIBCO) plus either 1μM rosiglitazone (Novo Nordisk) or vehicle (DMSO) for 2 days. After that, media was replaced for minimal maintenance media (Insulin/Rosi or Insulin/DMSO) for 2 days. At day 4 of differentiation proteins were lysed using radioimmunoprecipitation assay buffer (RIPA) (50mM Tris-HCl pH 7.6, 150mM NaCl, 0.1% sodium dodecyl sulfate, 0.5% sodium deoxycholate, 1% NP40 and freshly added halt protease/phosphate inhibitor cocktail (ThermoFisher). Immunoblots were performed using 20μg of protein. FABP4 was detected using rabbit FABP4 (Cell Signaling, 2120S), and VINCULIN as loading control (Sigma, V9131).

#### Polysome profile

Polysome profiles were obtained as described before.^92–94^ Briefly, stromal vascular fraction of *Lep*^*ob*^*/Lep*^*ob*^ mice was isolated, and primary cells were grown to confluency in 15-cm dishes. Cells were then treated to differentiate with complete adipogenic cocktail (0.5mM 3-isobutyl-1-methylxanthine (Sigma), 1μM dexamethasone (Sigma), 5μg/mL insulin (GIBCO) plus either 1μM rosiglitazone (Novo Nordisk) or vehicle (DMSO) for 2 days. After that, media was replaced for minimal maintenance media (Insulin/Rosi or Insulin/DMSO) for 2 days. At day 4 of differentiation cells were boosted with fresh media for one hour and for the last 5 minutes cycloheximide (Sigma) was added to the media (final concentration 100μg/mL). Cells were lysed and collected in hypotonic lysis buffer (5mM Tris HCl pH 7.5, 2.5 mM MgCl2, 1.5 mM KCl, 100 μg/mL cycloheximide, 2 mM DTT), and 0.5% Triton, 0.5% Sodium Deoxy-cholate were then added to the cell lysates after collection. The cytoplasmic extracts were resolved on 5–50% sucrose gradients by centrifugation in an SW40 rotor at 150,000 x g for 2 hours. The absorbance at 260 nm was measured using a Piston Gradient Fractionator Model 153 instrument (BioComp, Canada). The results were plotted in R as previously described.^95^

#### PPARγ ChIP-Seq

Publicly available dataset GSE41481 was downloaded from Gene Expression Omnibus.^51^ For each fat depot, sequenced reads from the ChIP-seq library were first aligned to mouse reference genome (mm9) with Bowtie2.^82^ Uniquely aligning read per genomic position was kept with Picard for downstream peak calling. Enriched PPARγ-binding regions were identified using HOMER^83^ findPeaks program (with “factor” mode) where default parameters for tag count normalization (to 10 million reads), false-discovery rate (FDR) cutoff (< 0.1%), and filtering options (≥ 4-fold tag count enrichment compared to both input control and local tag density) were applied. Peaks were annotated to the nearest TSS using HOMER annotatePeaks.pl program. Lastly, enriched peaks were visualized with Integrative Genomics Viewer (IGV).^96^

#### Total RNA sequencing and polysomes sequencing analysis

The RNA sample received was quantified using Qubit 2.0 Fluorometer (Life Technologies, Carlsbad, CA, USA) and RNA integrity was checked using TapeStation (Agilent Technologies, Palo Alto, CA, USA). The RNA sequencing library was prepared using the NEBNext Ultra RNA Library Prep Kit for Illumina using manufacturer’s instructions (NEB, Ipswich, MA, USA). Briefly, mRNAs were initially enriched with Oligod(T) beads. Enriched mRNAs were fragmented for 15 minutes at 94 °C. First-strand and second-strand cDNA were subsequently synthesized. cDNA fragments were end-repaired and adenylated at 3’ends, and universal adapters were ligated to cDNA fragments, followed by index addition and library enrichment by PCR with limited cycles. The sequencing library was validated on the Agilent TapeStation (Agilent Technologies, Palo Alto, CA, USA), and quantified by using Qubit 2.0 Fluorometer (Invitrogen, Carlsbad, CA) as well as by quantitative PCR (KAPA Biosystems, Wilmington, MA, USA). The sequencing library was clustered on three lanes of a flowcell. After clustering, the flowcell was loaded on the Illumina HiSeq instrument (4000 or equivalent) ac- cording to the manufacturer’s instructions. The sample was sequenced using a 2 × 150bp Paired End (PE) configuration. Image analysis and base calling were conducted by the HiSeq Control Software (HCS). Raw sequence data (.bcl files) generated from Illumina HiSeq was converted into fastq files and de-multiplexed using Illumina’s bcl2fastq 2.17 software. One mismatch was allowed for index sequence identification. Quality control of raw sequencing data was conducted using FastQC,^97^ employing default parameters. Subsequently, sequencing reads were mapped to the mouse genome mm10 using Salmon.^98^ Differential gene expression analysis was then undertaken with DESeq2.^84^ Genes meeting the criteria of an adjusted *p*-value less than 0.05 and a log2(fold change) greater than 0.2 were classified as differentially expressed. Pathway enrichment analysis was executed by examining the overlap between the identified DEGs and canonical pathways from databases such as KEGG, REACTOME, BIOCARTA, and HALLMARK using Fisher’s exact test. Multiple testing corrections were applied using the Benjamini-Hochberg method to determine the false discovery rate (FDR). Additionally, an enrichment score was calculated, capturing the proportion of overlapping genes in relation to the entire gene set specific to a given cell type. This score was normalized by the total number of genes detected in either total RNA sequencing or polysome sequencing.

#### qPCR

RNA from mAD/SVF or polysomes/total RNA pool was resuspended in Trizol reagent (ThermoFisher), and RNA was isolated using Direct-zol RNA Microprep kits as described by the manufacture (Zymo research). Isolated RNA was reverse transcribed using High-Capacity cDNA synthesis kit (ThermoFisher). Gene expression for selected genes was quantified using Quant Studio 6 Flex Real-Time PCR instrument, 384-well (Applied Biosystems by Invitrogen) with KAPA SYBR FAST qPCR 2x Master Mix Rox Low (Kapa Biosystems). A list of primer sequences can be found in Table S2.

#### Differentially translated transcripts analysis

Differential expression analysis was performed using DESeq2.^84^ We designed an interaction term between RNA fractions (i.e., polysome vs total RNA) and treatment (i.e., rosiglitazone vs vehicle) during DESeq2 analysis, which could normalize the expression level of total RNA abundance in polysome fractions. The transcripts with differential translation efficiency (DTETs) were defined using the criteria of adjusted *p*-values less than 0.05 and absolute log2(fold change) greater than 1. The upregulated transcripts after rosiglitazone treatments were classified as log2(fold change) greater than 1, while downregulated ones are classified as log2(fold change) less than −1.

#### Identification and quantification of enriched motifs

To explore the sequence patterns in upregulated transcripts, RNA sequences including 5’UTR, CDS, and 3’UTR were extracted based on the transcriptome annotations from GENCODE VM33. A statistic enrichment analysis was conducted to extract the enriched motifs from hexamer sequences in the upregulated transcripts regions and we chose the sequences from downregulated transcripts as the background group to measure the enrichment of hexamers. In brief, the sequences from the upregulated transcripts regions were extracted by overlapping hexamers using the window length as 6nt and step size as 1nt. The occurrences and frequencies of each hexamer were counted across sequences and the enrichment score was calculated using Z-test. The enriched hexamers between up- vs downregulated transcripts were defined with the enrichment z-scores larger than 3. The motif analyses are performed using RSAT.^85^ First, the position-specific scoring matrix (PSSM) of the enriched hexamers was obtained using the ‘convert-matrix’ program. Sequence motifs were generated using R package ‘ggseqlogo’. Then, we scanned the enriched 5’UTR motif across each position of the 5’UTR in upregulated transcripts using the ‘matrix-scan’ program, which is a pattern-matching method calculating the similarity between sequences with PSSM. The 5’UTR sites with *p*-values lower than 0.05 were selected as the possible regulatory sites to generate a feature map.

#### Design of truncated 5’UTRs

All significant motif sites were used to design the truncated 5’UTRs. We scanned each 5’UTR using the sliding windows in 100nt length with 1nt step size and then counted all the significant motif sites in each 100nt sliding window. The windows with more than 1 motif site were defined as potential functional windows. If there were at least two functional windows in adjacent positions, the windows were merged into a longer one which was defined as the candidate truncated 5’UTRs.

#### eIF4A inhibition

The C3H/10T1/2 cell line was obtained from ATCC (Clone 8, CL-226). 10T1/2 cells were subjected to limiting dilution to identify a monoclonal cell population exhibiting high adipogenic potential (based on lipid droplet formation and PPARγ target gene expression). 10T1/2 clone#22 cells were gifted from Tontonoz Lab. 10T1/2 clone#22 cells were cultured in DMEM (GIBCO), 10% FBS (Omega FB#11) until confluency and subjected to adipogenic differentiation with DMI+GW: 5μg/mL insulin (GIBCO), 0.5mM 3-isobutyl-1-methylxanthine (Sigma), 1μM dexamethasone (Sigma), and 20nM GW1929 (Cayman). After 24 hours, the induction media (DMI+GW) was replaced with the addition of 0.25–25nM of CR-1–31-B (MedChemExpress). After 24h, the media was replaced my maintenance media (DMEM, insulin, GW) with the addition of 0.25–25nM of CR-1–31-B, totalizing a 48h treatment with CR-1–31-B. For the experiments with primary cells, cells were isolated and sorted as previously described. Briefly, DPP4+ and DPP4- cells from ob/ob Veh or Rosi mice were plated in a 96-well plate and culture in DMEM (GIBCO), 5μg/mL insulin (GIBCO), 10% FBS (Omega FB#11), 1% PenStrep (GIBCO), and 50μg/mL Primocin (Invivogen) until confluency (24h). Subsequently, the media was replaced with DMEM, 10% FBS, insulin and 10nM of CR-1–31-B for 48h. Cells were then fixed, stained, and imaged as previously described.

#### ChIP qPCR

ChIP was performed following a previously published protocol with minor modifications.^99^ Briefly, 10T1/2 and 3T3-L1 were differentiated for 5 days with DMI+GW. Crosslinking was performed with 2 mM DSG/PBS RT for 60 min, followed by PBS containing 1% methanol free formaldehyde, RT for 11 min. 40 million cells were lysed and sonicated using M220 Focused-ultra sonicator (Covaris) according to the manufacturer’s protocol. Chromatin was immunoprecipitated with 5 mL antibodies against PPARγ (81B8) (Cell Signaling Technology) or IgG (Millipore) overnight at 4°C. After reversal of cross-linking, DNA was isolated, and DNA enrichment was quantified by q-PCR. Agpat2 was used as positive control of PPARγ ChIP-qPCR. The sequence of primers used for ChIP-qPCR is provided in Table S2.

#### Proteomics and identification of differentially expressed proteins

The cells treated with either Veh or Rosi were lysed in RIPA buffer as previously described and protein concentration was estimated using BCA assay. 50mg total protein was reduced and alkylated using 5μM TCEP (tris-2-carboxyethyl phosphine) and 10mM iodoacetamide, respectively. This was followed by protein clean-up using SP3 protocol^100^ before subjecting to overnight digestion with a mixture of trypsin and lysC proteases. The peptide digests were acidified with formic acid and cleaned using SP3 peptide clean-up protocol.^100^ The dried peptides were resuspended in 5% formic acid and analyzed using LC-MS/MS on a Bruker timsTOF HT instrument. On-line peptide fractionation was performed on an ionopticks AuroraElite C18 Column (75μm ID, 15 cm length, 1.5 μm particle size) connected to a Vanquish Neo UHPLC-System (Thermo Scientific) operated at a flow rate of 500nl/min. The data acquisition mode was selected as diaPASEF ^101^ with m/z window width set to 10m/z covering m/z range of 300–1200, mobility range set to 0.60–1.60 Vs cm^−2^, 100ms ramp time and 50ms accumulation time. DIA-NN software was used for database searching of the raw data with an *in-silico* library generated from *Mus musculus* database.^102^ DIANN output was analyzed on Fragpipe-analyst, resulting in the identification of differentially regulated proteins.^103^ Proteins with adjusted *p*-value < 0.05 and |log2FC| > 2 were identified as being significantly regulated by Rosiglitazone treatment. Volcano plots of these differentially expressed proteins were derived with ggplot2 R package. Significant DE ribosomal proteins were defined with cutoffs of adjusted *p*-value < 0.05 and |log2FC| > 0.25.

### QUANTIFICATION AND STATISTICAL ANALYSIS

For graphs, data are shown as mean ± SEM. GraphPad (GP) pvalue style: *p = 0.0332; **p < 0.0021; ***p < 0.0002. Statistical differences were evaluated using Student t-test or otherwise state in the legends of the figures. Graphs were produced and statistical analyses were performed using GraphPad Prism.

## Supplementary Material

1

2

3

4

## Figures and Tables

**Table:** KEY RESOURCES TABLE

REAGENT or RESOURCE	SOURCE	IDENTIFIER

**Antibodies**		

Pacific Blue anti-mouse CD45.2 (104)	BioLegend	Cat# 109819;RRID:AB_492873
PE-Cy7 anti-mouse CD45.2 (104)	BioLegend	Cat# 109830; RRID:AB_1186098
APC/Cy7 anti-mouse TCRβ (H57-597)	BioLegend	Cat# 109220; RRID:AB_893624
APC/Cy7 anti-mouse CD3 (17A2)	BioLegend	Cat# 100222;RRID:AB_2242784
APC/Cy7 anti-mouse CD19 (6D5)	BioLegend	Cat# 115529;RRID:AB_830706
APC/Cy7 anti-mouse NK1.1 (PK136)	BioLegend	Cat# 108723;RRID:AB_830870
APC/Cy7 anti-mouse Ly6G (1A8)	BioLegend	Cat# 127623;RRID:AB_10645331
PE anti-mouse CD11c (N418)	BioLegend	Cat# 117307;RRID:AB_313776
FITC anti-mouse CD11b (M1/70)	BioLegend	Cat# 101205;RRID:AB_312788
APC anti-mouse CD88 (20/70)	BioLegend	Cat# 135808;RRID:AB_10899415
PerCP/Cy5.5 anti-mouse CD9 (MZ3)	BioLegend	Cat# 124817;RRID:AB_2783076
PE-Cy7 anti-mouse Tim-4 (RMT4-54)	BioLegend	Cat# 130009;RRID:AB_2565718
Pacific Blue anti-mouse CD31 (390)	BioLegend	Cat# 102421;RRID:AB_10613457
APC anti-mouse CD140a (PDGFRα) (APA5)	BioLegend	Cat# 135907;RRID:AB_2043969
PerCP-Cy5.5 anti-mouse CD26 (DPP4) (H194-112)	eBioscience	Cat# 45-0261-82;RRID:AB_1548738
PE anti-mouse CD54 (ICAM-1) (YN1/1.7.4)	eBioscience	Cat# 12-0541-81;RRID:AB_465706
Alexa Fluor 488 anti-mouse CD36 (HM36)	BioLegend	Cat# 102607;RRID:AB_528791
Rabbit anti-PLIN1	Abcam	Cat# 3526;RRID:AB_2167274
Rat anti-F4/80 (BM8)	BioLegend	Cat# 123101;RRID:AB_893504
Rabbit anti-Fabp4	Cell Signaling	Cat# 2120S
Mouse anti-Vinculin	Sigma	Cat# V9131;RRID:AB_477629
Mouse anti-Puromycin	Sigma	Cat# 12D10
HRP Goat anti-mouse IgG, Fcy subclass 2a	Jackson Immuno Research	Cat# 115-035-206;RRID:AB_2338514
Donkey anti-rat IgG Alexa Fluor 568	Abcam	Cat# 175475;RRID:AB_2636887
Donkey anti-rabbit IgG Alexa Fluor 647	Abcam	Cat# 150063;RRID:AB_2687541

**Chemicals, peptides, and recombinant proteins**		

CR-1-31-B	MedChemExpress	Cat# HY-136453
Rosiglitazone (*in vitro*)	Cayman	Cat# 71740
Rosiglitazone (*in vivo*)	Sigma	Cat# R2408
Insulin	Gibco	Cat #12585014
Dexamethasone	Sigma	Cat# D4902
3-isobutyl-1-methylxanthine	Sigma	Cat# I5879
LipidTox Green Neutral Lipid	Invitrogen	Cat# H34475
DAPI	AAT Bioquest	Cat# 17507
Type II Collagenase	Worthington	Cat# LS004176
Type II Collagenase	Sigma	Cat# C6885
2.6% Methylcellulose	StemCell Technologies	Cat# M3120

**Deposited data**		

scRNA-Seq	Gene Expression Omnibus	GSE267192
Polysome sequencing	Gene Expression Omnibus	GSE268057

**Experimental models: Cell lines**		

C3H/10T1/2 clone#22	ATCC- clonal selection Tontonoz Lab	Clone#8, CL-226
C3H/10T1/2	ATCC	Clone#8, CL-226
3T3-L1	ATCC	CL-173

**Experimental models: Organisms/strains**		

Mouse C56BL/6	The Jackson Laboratory	JAX:000664
Mouse Lep^ob^/Lep^ob^	The Jackson Laboratory	JAX:000632

**Oligonucleotides**		

qPCR primers, see Table S2	This paper	N/A

**Software and algorithms**		

GraphPad Prism 10	GraphPad	https://www.graphpad.com/
ImageJ	National Institutes of Health	https://imagej.net/ij/
FlowJo, Version 10.10.0	Ashland, OR: Becton, Dickinson and Company	https://www.flowjo.com/solutions/flowjo
R and RStudio	R Consortium	https://posit.co/products/open-source/rstudio/
Seurat	Butler et al.^43^	https://satijalab.org/seurat/
Bowtie2	Langmead and Salzberg^82^	https://bowtie-bio.sourceforge.net/bowtie2/index.shtml
Picard	Broad Institute	https://broadinstitute.github.io/picard/
HOMER	Heinz et al.^83^	http://homer.ucsd.edu/homer/
DESeq2	Love et al.^84^	https://bioconductor.org/packages/release/bioc/html/DESeq2.html
RSAT	Turatsinze et al^85^	https://rsat.france-bioinformatique.fr/metazoa/index.php
ggseqlogo	Wagih et al^86^	https://cran.r-project.org/web/packages/ggseqlogo/index.html
10X Genomics Cell Ranger version 3.0.2	Zheng et al^87^	https://www.10xgenomics.com/cn/support/software/cell-ranger/

## Highlights

- Single-cell RNA sequencing reveals reprogramming of adipocyte precursors
- PPARγ agonist stimulates ribosomal gene expression in adipocyte precursors in obesity
- Polysome profiling of adipocytes shows translational selectivity of mRNAs
- Inhibition of translation with eIF4A inhibitor blocks adipogenesis
