# Supplementary material for: PPARγ-dependent remodeling of translational machinery in adipose progenitors is impaired in obesity
Source: Cell Rep. Author manuscript; Available in PMC 2025 Apr 16. (PMC12002411; doi:10.1016/j.celrep.2024.114945)
Supplement: 1 [file NIHMS2044431-supplement-1.pdf]

Cell Reports, Volume *43*

Supplemental information

PPARγ-dependent remodeling of translational machinery in adipose progenitors is impaired in obesity

Mirian Krystel De Siqueira, Gaoyan Li, Yutian Zhao, Siqi Wang, In Sook Ahn, Mikayla Tamboline, Andrew D. Hildreth, Jakeline Larios, Alejandro Schcolnik-Cabrera, Zaynab Nouhi, Zhengyi Zhang, Marcus J. Tol, Vijaya Pandey, Shili Xu, Timothy E. O'Sullivan, Julia J. Mack, Peter Tontonoz, Tamer Sallam, James A. Wohlschlegel, Laura Hulea, Xinshu Xiao, Xia Yang, and Claudio J. Villanueva

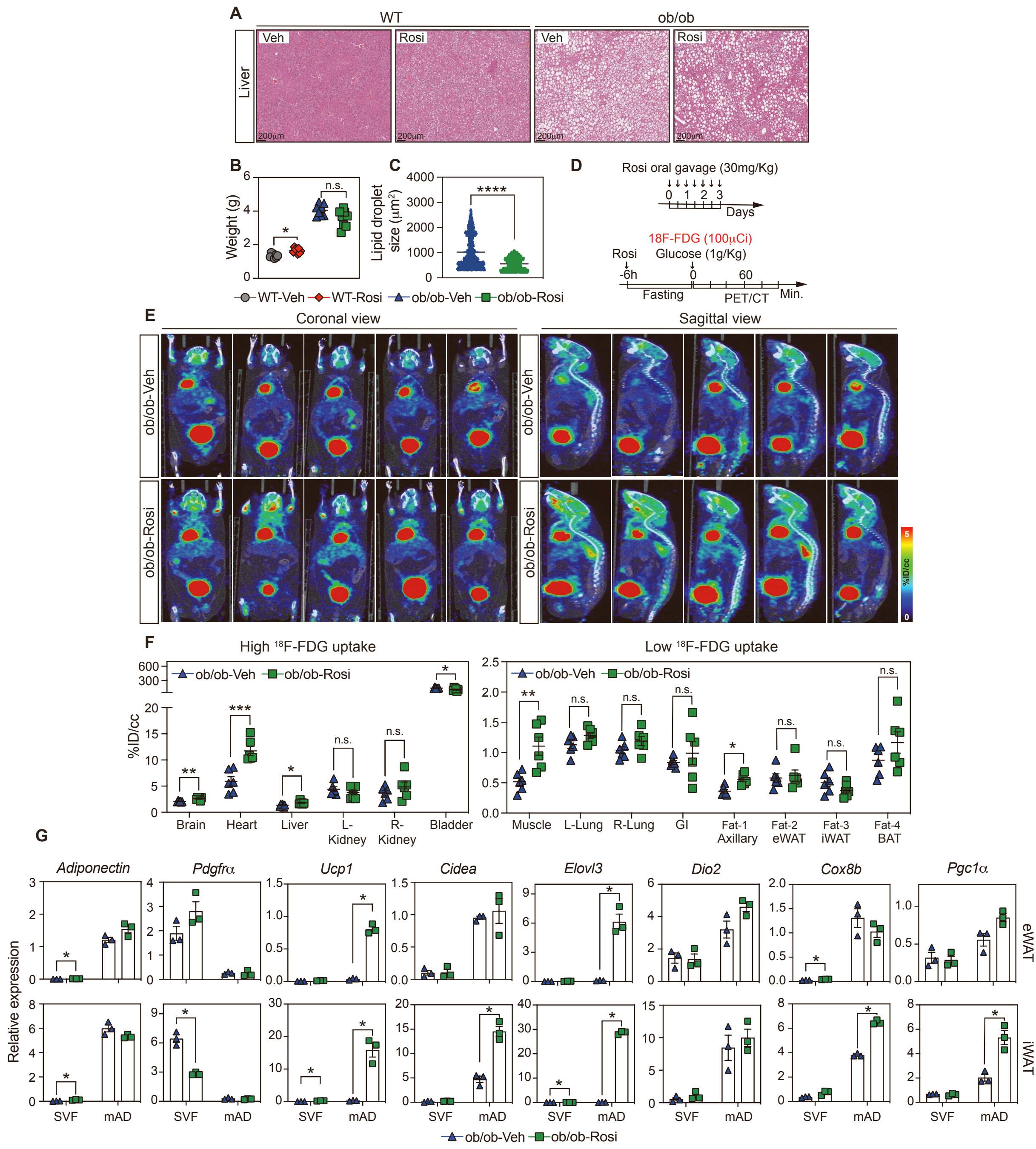

Fig.S1| Host physiology remodeling after Rosiglitazone treatment. (**A**) Histological analysis of the liver after acute Rosi treatment (30mg/Kg). (**B**) Liver weight. (**C**) Lipid droplet area quantification of ob/ob mice treated with either Veh or Rosi. (**D**) Schematic overview of conscious glucose uptake in vivo using a bolus of glucose (1g/Kg) and [^{18}F]-FDG (100µCi). (**E**) PET/CTs at coronal and sagittal view of ob/ob mice treated either Veh or Rosi. (**F**) Quantification of percent injected dose per cubic centimeter in tissue (%ID/cc) ID, injected dose. Data represent mean ± SEM (n = 6-8 mice per group). (**G**) RT-PCR from isolate SVF and mature adipocytes (mAD) from eWAT and iWAT ob/ob mice treated with either Veh or Rosi (n=3). GraphPad (GP) pvalue style: *p= 0.0332; **p < 0.0021; ***p < 0.0002 by (B) one-way ANOVA, multiple comparisons followed

by Tukey post hoc test, (C) two-tailed Student’s t-test, and (F-G) multiple comparisons two-tailed Student’s t-test.

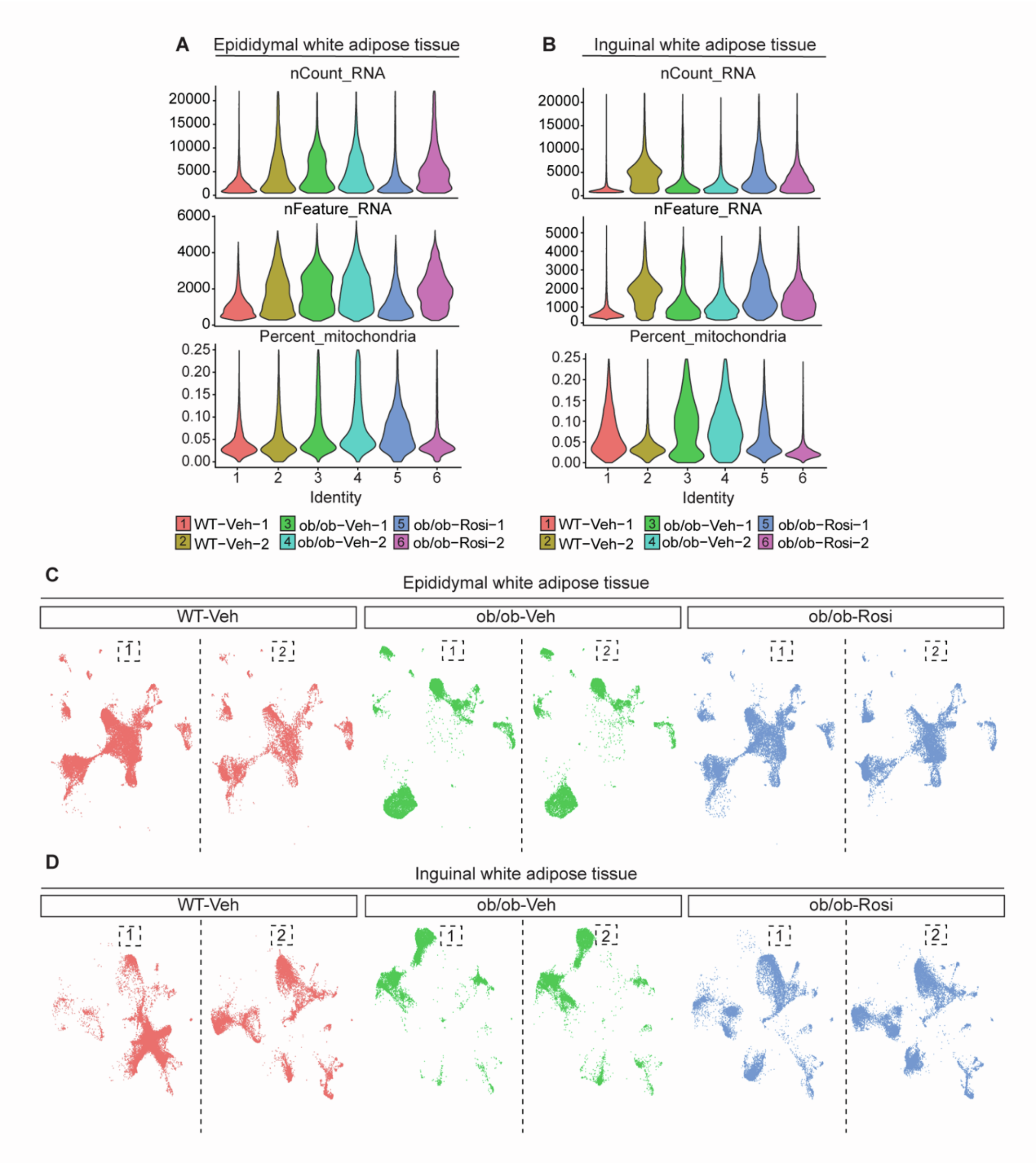

Fig.S2| Adipose stromal vascular fraction Single-Cell RNA Sequencing quality control. (**A-B**) Violin plots display the distribution of the number of Unique Molecular Identifiers (UMI) (nCount_RNA), the number of detected genes (nFeature_RNA), and the proportion of mitochondrial reads (Percent_mitochondria) across each sample in eWAT (**A**) and iWAT (**B**). Cells were selected based on the number of UMIs (with a threshold range of 700-22,000), the number of detected genes (with a threshold range of 200-6,000), and the proportion of mitochondrial reads (with a threshold of less than 25%). (**C-D**) UMAP plot illustrates the cell clusters among 61,343 eWAT cells (**C**) and 65,556 iWAT cells (**D**) by each sample. The three panels separately represent cells from WT-Veh, ob/ob-Veh, and ob/ob-Rosi groups. Each colored dot signifies a cell. Data shown a single scRNA-Seq experiment (n=2 per group).

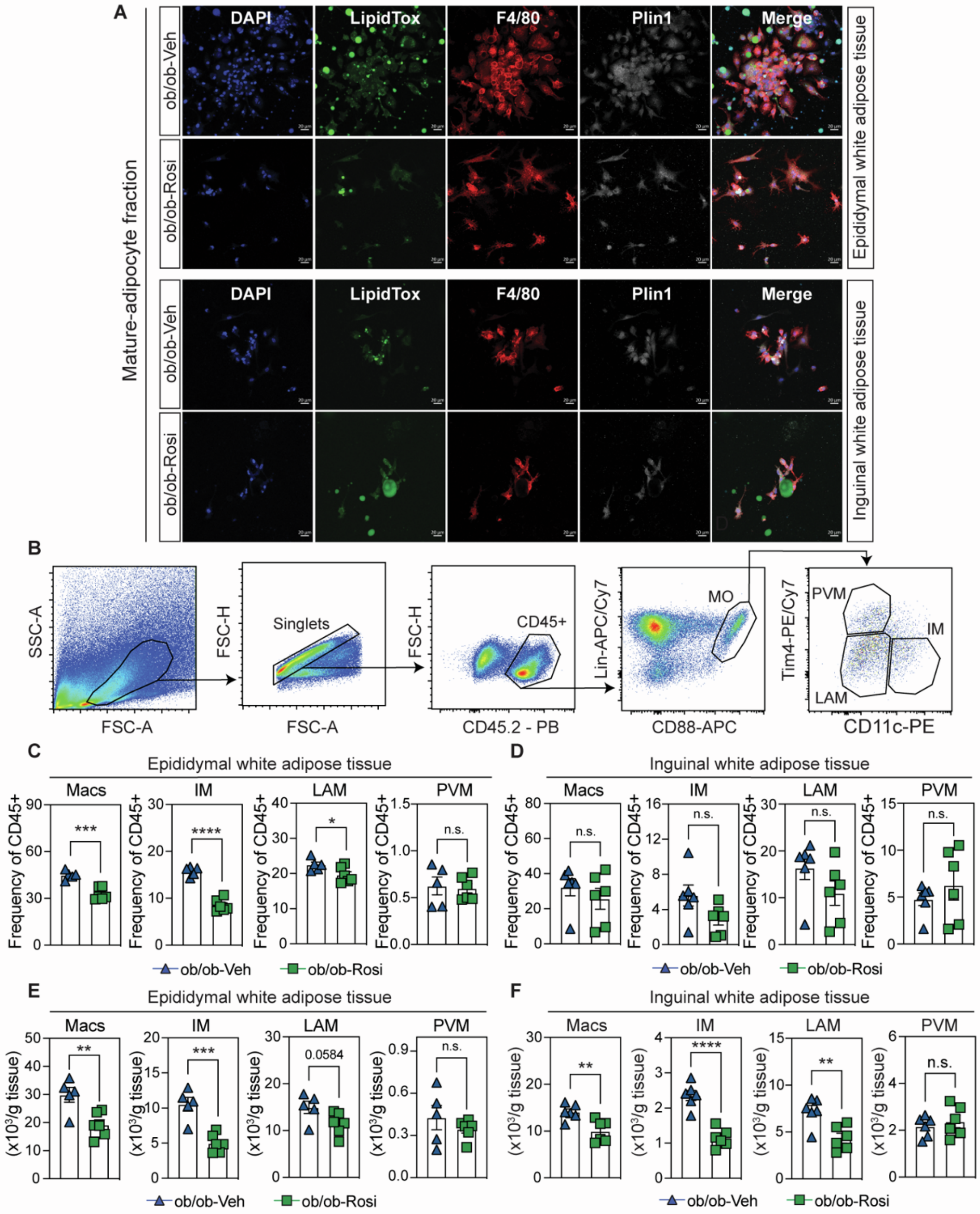

Fig.S3| Adipose-specific macrophage profile remodeling after PPARγ agonist treatment. (**A**) Confocal images of macrophages (F4/80+) cultured overnight from mature-adipocyte fraction from either ob/ob-Veh or ob/ob-Rosi: DAPI (nuclei- blue), LipidTox (neutral lipids-green), F4/80 (macrophages-red), Plin1 (adipocyte-specific perilipin - white). (**B**) Flow cytometry gate strategy for macrophages: Tim4+: perivascular macrophages (PVM), CD11c+: inflammatory macrophages (IM), Tim4-, CD11c-: lipid-associated macrophages (LAM). (**C-F**) Frequency and absolute number of Macrophages (Mac), IM, LAM, and PVM from epididymal and inguinal adipose tissue. Data represent mean ± SEM (n = 6 mice per group). GraphPad (GP) pvalue style: *p= 0.0332; **p < 0.0021; ***p < 0.0002 by two-tailed Student's t-test.

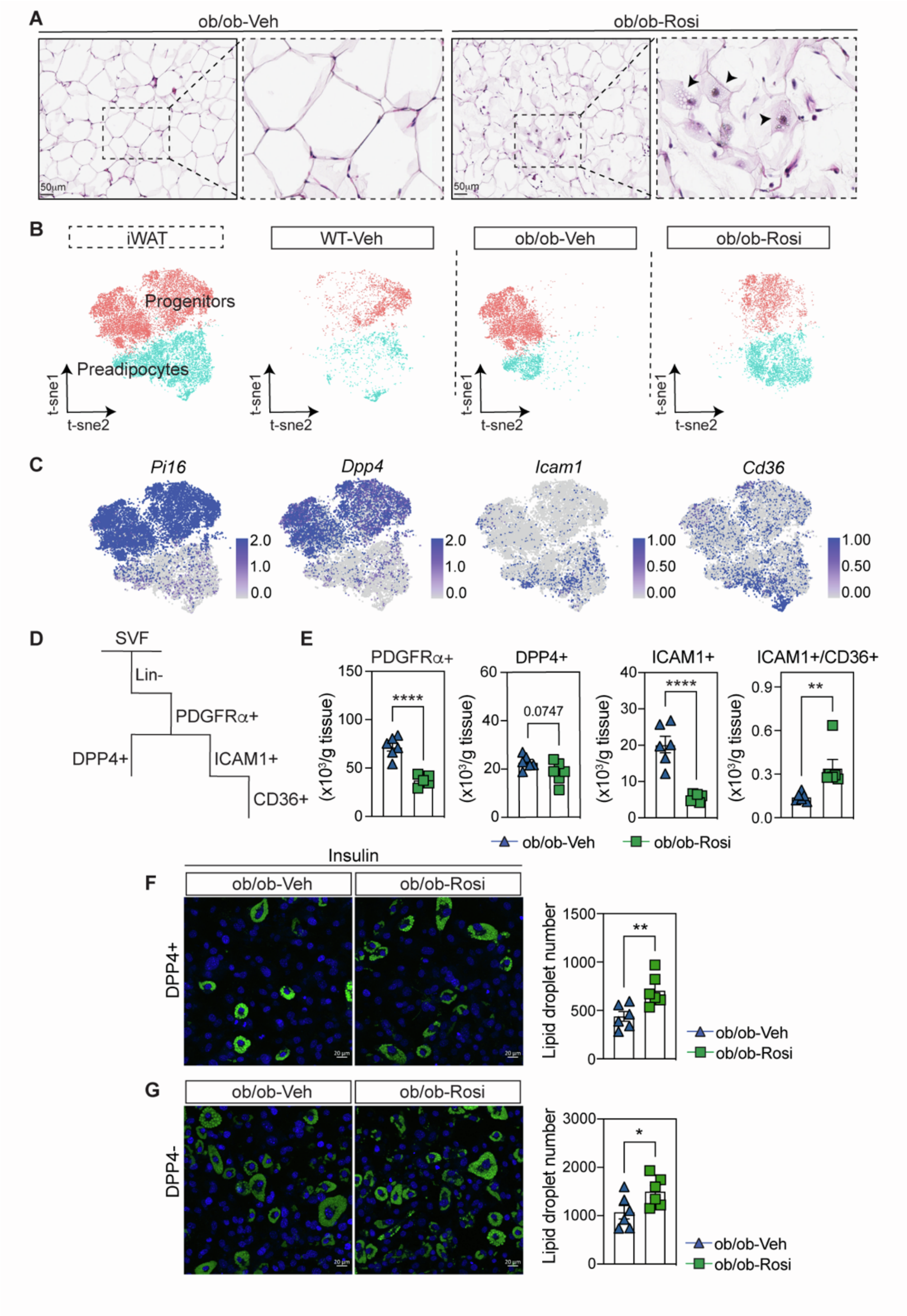

Fig. S4| Rosiglitazone-driven enhancement of adipocyte differentiation in inguinal adipose tissue. (**A**) Histological analysis of inguinal adipose tissue (hematoxylin and eosin stain) from either ob/ob-Veh or ob/ob-Rosi mice. (**B**) t-distributed stochastic neighbor embedding (t-SNE) plot illustrates two sub-clusters of Adipocyte Precursor Cells (APC) in iWAT: Progenitors and preadipocytes. The right three panels separately represent cells from WT-Veh, ob/ob-Veh, and ob/ob-Rosi. Each color-coded dot represents a cell, with progenitors being represented by red and preadipocytes by cyan. The Louvain algorithm was utilized to determine cell clusters. (**C**) Individual gene t-SNE plots showing the expression and distribution of representative marker genes: *Pi16* and *Dpp4* for progenitors, *Icam1* and *Cd36* for preadipocytes. (**D**) Gate strategy to

characterize progenitor cells: Lineage negative (CD45-, CD31-), PDGFRα+, DPP4+ (progenitors), and ICAM1+/CD36+ (preadipocytes). (**E**) Absolute number of progenitor and preadipocytes from ob/ob-Veh and ob/ob-Rosi mice. (**F-G**) Confocal of sorted lineage negative (CD45-, CD31-), PDGFRα+, DPP4+, and DPP4- cells differentiated for 4 days on insulin-containing media, DAPI (nuclei-blue), LipidTox (neutral lipids-green). Data represent mean ± SEM (n = 3 mice per group). Confocal images: 4 wells per condition, 2 representative images per well were acquired. GraphPad (GP) pvalue style: *p= 0.0332; **p < 0.0021; ***p < 0.0002 by two-tailed Student's t-test.

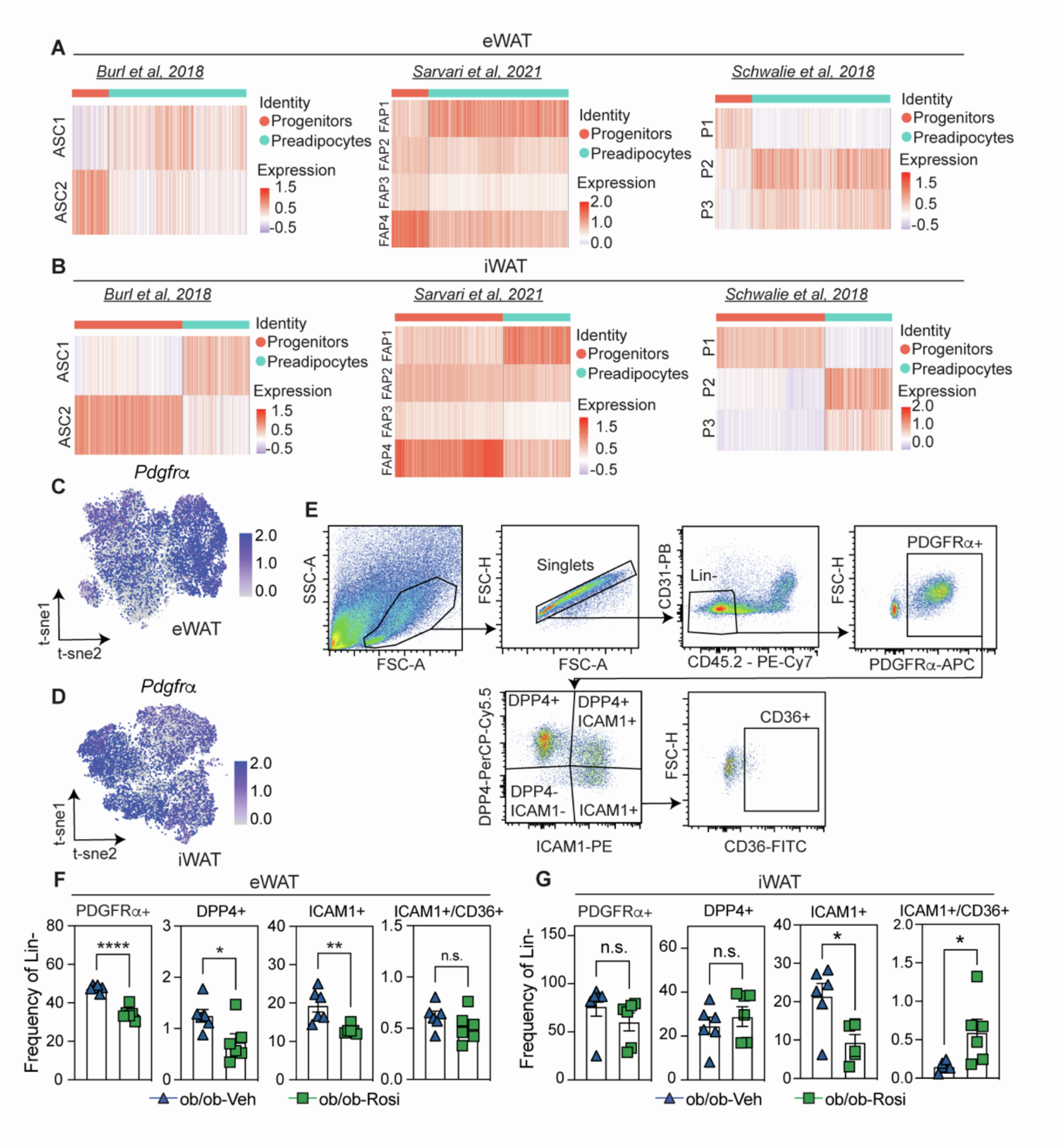

Fig.S5| Validation of progenitor and preadipocyte populations (A-B) Heatmap illustrates gene module scores within eWAT (**A**) and iWAT (**B**) cells by APC subtype identities. The gene module scores feature the top 50 significant markers from each reference cell type, as delineated in the research findings of Burl *et al*., Sarvari *et al*., and Schwalie *et al*. (**C-D**) t-SNE visualization of *Pdgfrα* expression in eWAT and iWAT cells respectively. (**E**) Flow cytometry gate strategy for progenitors and preadipocytes: Lin- (CD45-, CD31-), PDGFRα+, DPP4+ (progenitors), and ICAM1+ and/or ICAM1+/CD36+ (preadipocytes). (**F-G**) Frequency of progenitors and preadipocytes from epididymal and inguinal adipose tissue. Data represent mean ± SEM (n = 6 mice per group). GraphPad (GP) pvalue style: *p= 0.0332; **p < 0.0021; ***p < 0.0002 by two-tailed Student's t-test.

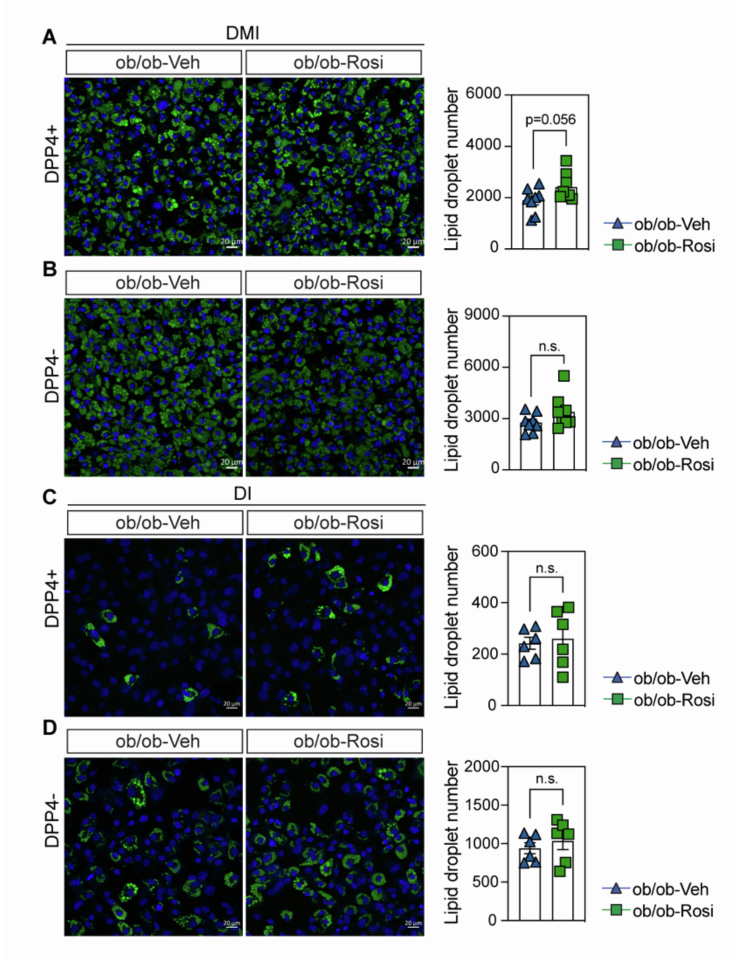

Fig.S6| Inguinal adipose tissue progenitors differentiation potential after Rosiglitazone treatment. (**A**-**D**) Confocal of sorted lineage negative (CD45-, CD31-), PDGFRα+, DPP4+, and DPP4- cells differentiated for 4 days on DMI (dexamethasone, IBMX, and insulin) media, DAPI (nuclei- blue), LipidTox (neutral lipids- green) (**A-B**). 4 days on DI (dexamethasone, and insulin) media, DAPI (nuclei- blue), LipidTox (neutral lipids- green) (**C-D**). Right panels: quantification of lipid droplet number. Data represent mean ± SEM (n = 3-6 mice per group). Confocal images: 4 wells per condition, 2 representative images per well were acquired. GraphPad (GP) pvalue style: *p= 0.0332; **p < 0.0021; ***p < 0.0002 by two-tailed Student's t-test.

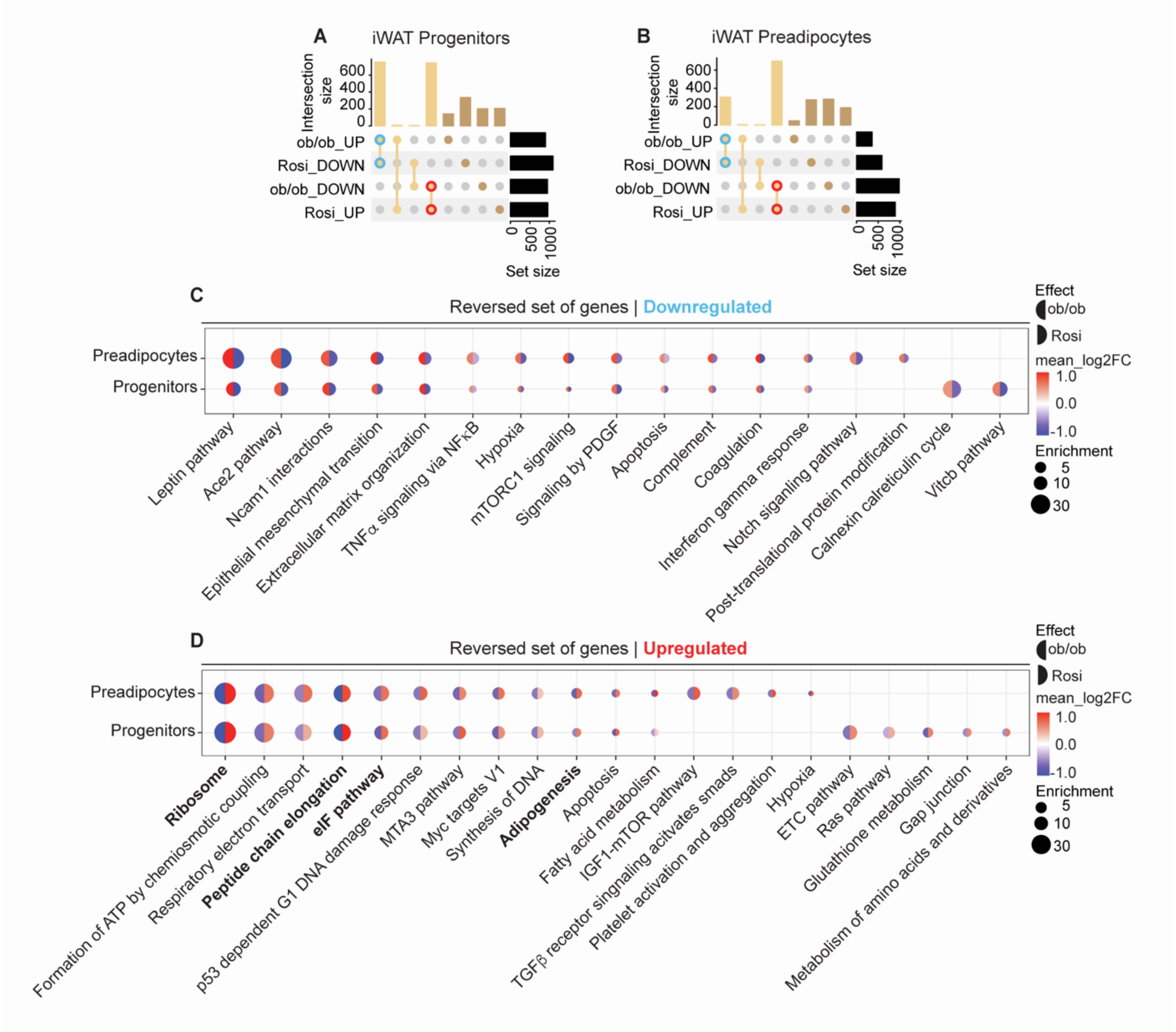

Fig. S7| Comparison of differently expressed and enriched pathways in response to obesity and Rosiglitazone treatment in the iWAT (**A-D**) UpSet plot illustrates intersections of differentially expressed genes (DEGs) for the iWAT progenitors (**A**), and iWAT preadipocytes (**B**), all at Benjamini-Hochberg adjusted p-value < 0.05. The 4 categories include upregulated DEGs in obese mice compared to lean mice (ob/ob_UP), downregulated DEGs in obese mice compared to lean mice (ob/ob_DOWN), up-regulated DEGs in response to Rosi treatment compared to ob/ob-Veh (Rosi_UP), and downregulated DEGs in response to Rosi treatment compared to ob/ob-Veh (Rosi_DOWN). Horizontal bars (set size) indicate total DEGs for each cluster in each plot. In the UpSet plots, dots point to the specific clusters for which the vertical bars for DEG counts are shown, and vertical lines between dots represent the intersections between two or more clusters. The blue circle signifies the set of DEGs that are upregulated in obese mice and downregulated in response to Rosi treatment. The red circle represents the DEGs that are downregulated in obese mice and upregulated following Rosi treatment. (**C-D**) Dot plot illustrates the top enriched pathways in response to Rosi treatment, which acts to reverse the effects of obesity. All pathways displayed meet the cut-off for statistical significance at Benjamini-Hochberg adjusted p values < 0.05. The size of each dot corresponds to the enrichment score for each pathway, reflecting the ratio of overlapping genes to total genes within the cell type-specific gene set, adjusted by a scale factor of 20,000, and then divided by the total number of genes within the

pathway. Color of the left side of each dot represents the log2(fold-change), calculated based on the average fold change across all overlapping DEGs within a pathway in obese mice compared to lean mice. Color of the right side of each dot represents the log2 (fold-change), calculated based on the average fold change across all overlapping DEGs within a pathway in response to Rosi treatment. (**C**) Represented pathways enriched from DEGs that are upregulated in iWAT obese mice and downregulated following Rosi treatment. (**D**) Represented pathways enriched from DEGs that are downregulated in iWAT obese mice and upregulated following Rosi treatment.

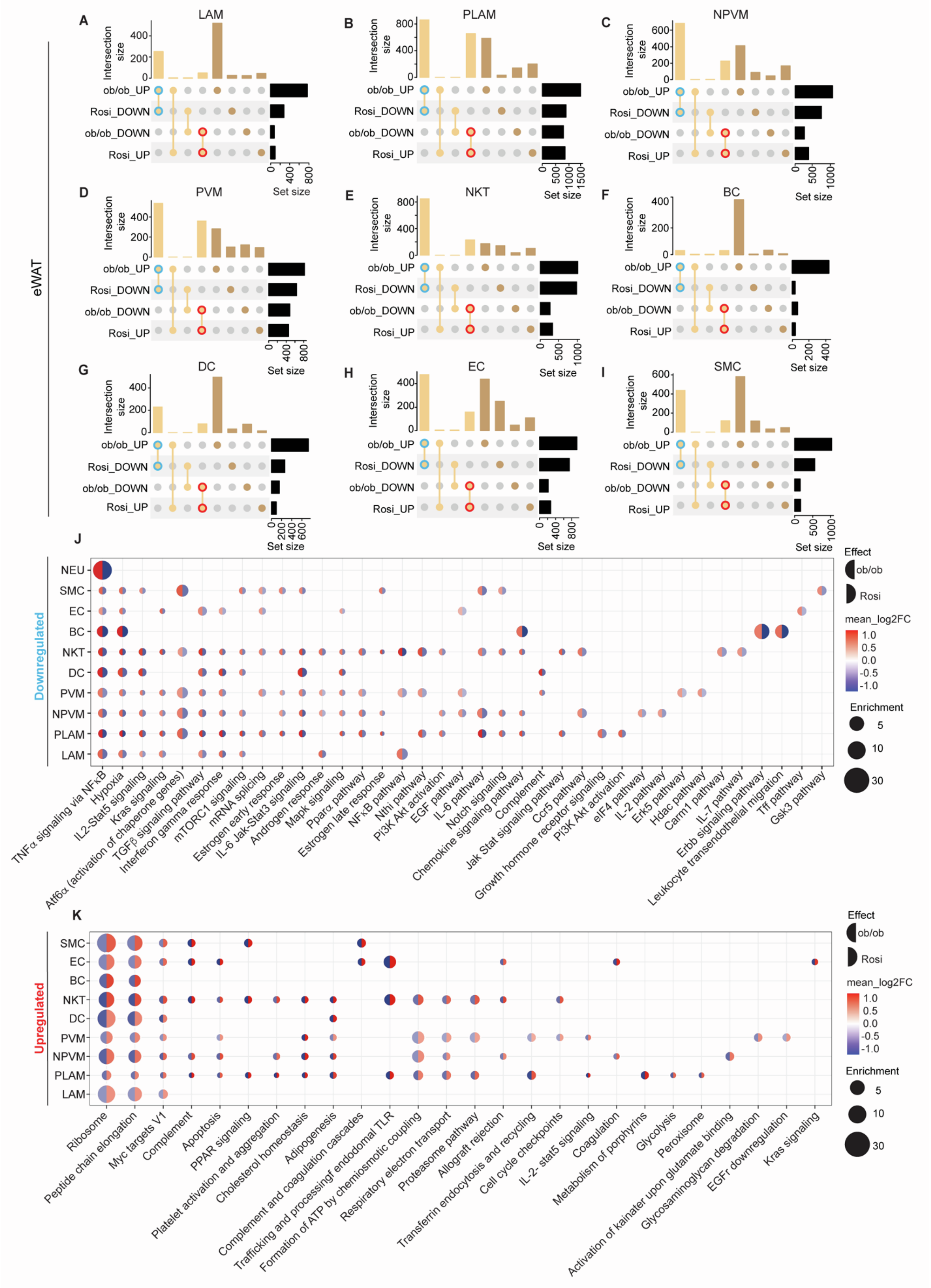

A
LAM
B
PLAM
C
NPVM
D
PVM
E
NKT
F
BC
G
DC
H
EC
I
SMC
eWAT
Intersection size
Set size
ob/ob_UP
Rosi_DOWN
ob/ob_DOWN
Rosi_UP
J
Downregulated
NEU
SMC
EC
BC
NKT
DC
PVM
NPVM
PLAM
LAM
TNFα signaling via NFκB
Hypoxia
IL2-Stat5 signaling
Kras signaling
Atf6α (activation of chaperone genes)
TGFβ signaling pathway
Interferon gamma response
mTORC1 signaling
mRNA splicing
Estrogen early response
IL-6 Jak-Stat3 signaling
Androgen response
Mapk signaling
Pparα pathway
Estrogen late response
NFκB pathway
Nthi pathway
Pi3K Akt activation
EGF pathway
IL-6 pathway
Notch signaling
Chemokine signaling pathway
Complement
Jak Stat signaling pathway
Ccr5 pathway
Growth hormone receptor signaling
Pi3K Akt activation
eIF4 pathway
IL-2 pathway
Erk5 pathway
Hdac pathway
Carm1 pathway
IL-7 pathway
Erbb signaling pathway
Leukocyte transendothelial migration
Tff pathway
Gsk3 pathway
Effect
ob/ob
Rosi
mean_log2FC
Enrichment
K
Upregulated
Ribosome
Peptide chain elongation
Myc targets V1
Complement
Apoptosis
PPAR signaling
Platelet activation and aggregation
Cholesterol homeostasis
Adipogenesis
Complement and coagulation cascades
Trafficking and processingf endodomal TLR
Formation of ATP by chemiosmotic coupling
Respiratory electron transport
Proteasome pathway
Allograft rejection
Transferrin endocytosis and recycling
Cell cycle checkpoints
IL-2- stat5 signaling
Coagulation
Metabolism of porphyrins
Glycolysis
Peroxisome
Activation of kainater upon glutamate binding
Glycosaminoglycan degradation
EGFr downregulation
Kras signaling

Fig.S8| Effects of Rosiglitazone treatment in the stromal vascular fraction of epididymal adipose tissue. (**A-I**) UpSet plot illustrates intersections of DEGs for the cell types in eWAT. (**J-K**) Dot plot illustrates the top enriched pathways in response to Rosi treatment in each cell type, which acts to reverse the effects of obesity. (**J**) represent pathways enriched from DEGs that are upregulated in obese mice and downregulated following Rosi treatment and (**K**) represent pathways enriched from DEGs that are downregulated in obese mice and upregulated following Rosi treatment.

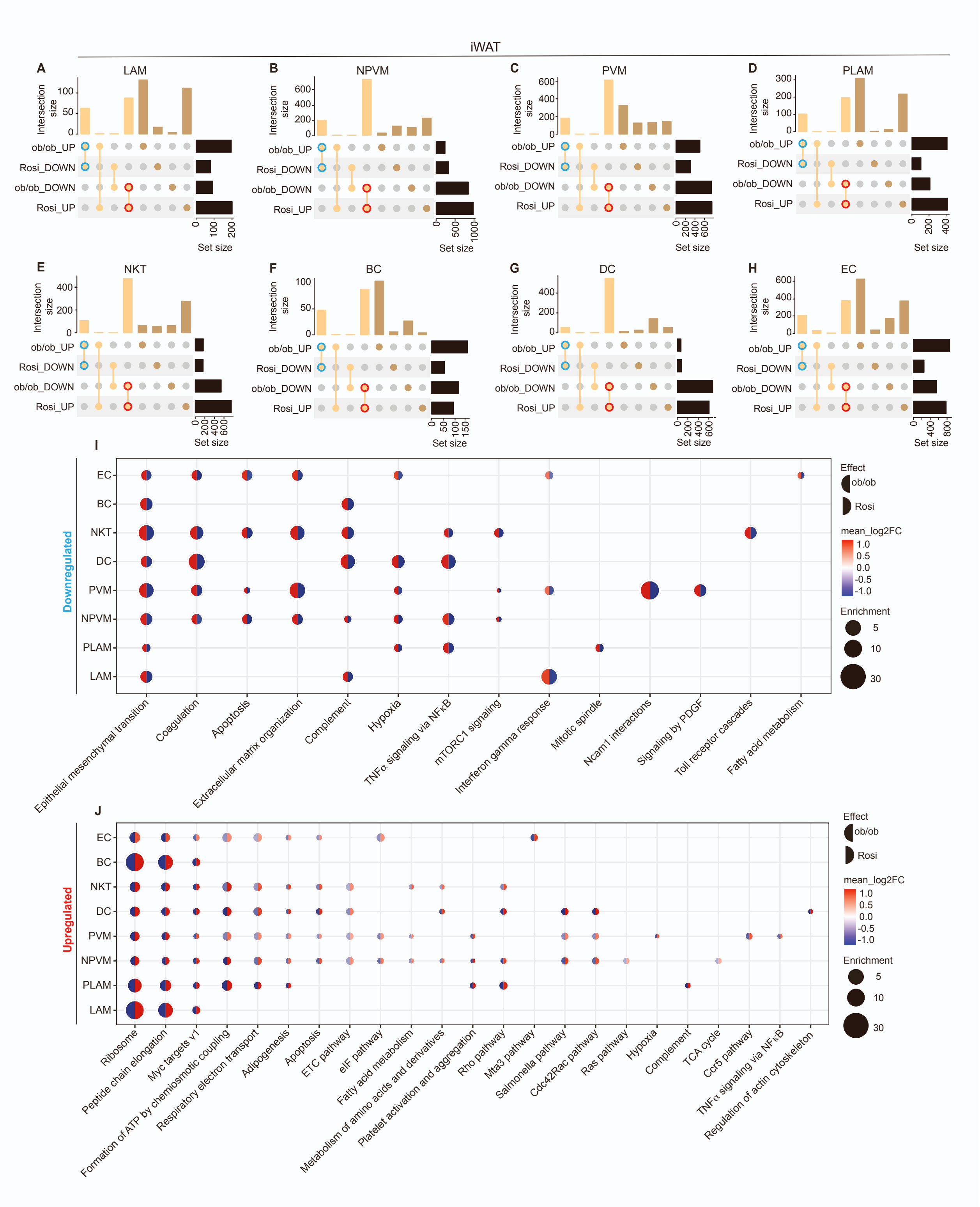

Fig.S9| Effects of Rosiglitazone treatment in the stromal vascular fraction of inguinal adipose tissue. (**A-H**) UpSet plot illustrates intersections of DEGs for the cell types in iWAT. (**I-J**) Dot plot illustrates the top enriched pathways in response to Rosi treatment in each cell type,

which acts to reverse the effects of obesity. (**I**) represent pathways enriched from DEGs that are upregulated in obese mice and downregulated following Rosi treatment. Conversely, (**J**) represents pathways enriched from DEGs that are downregulated in obese mice and upregulated following Rosi treatment.

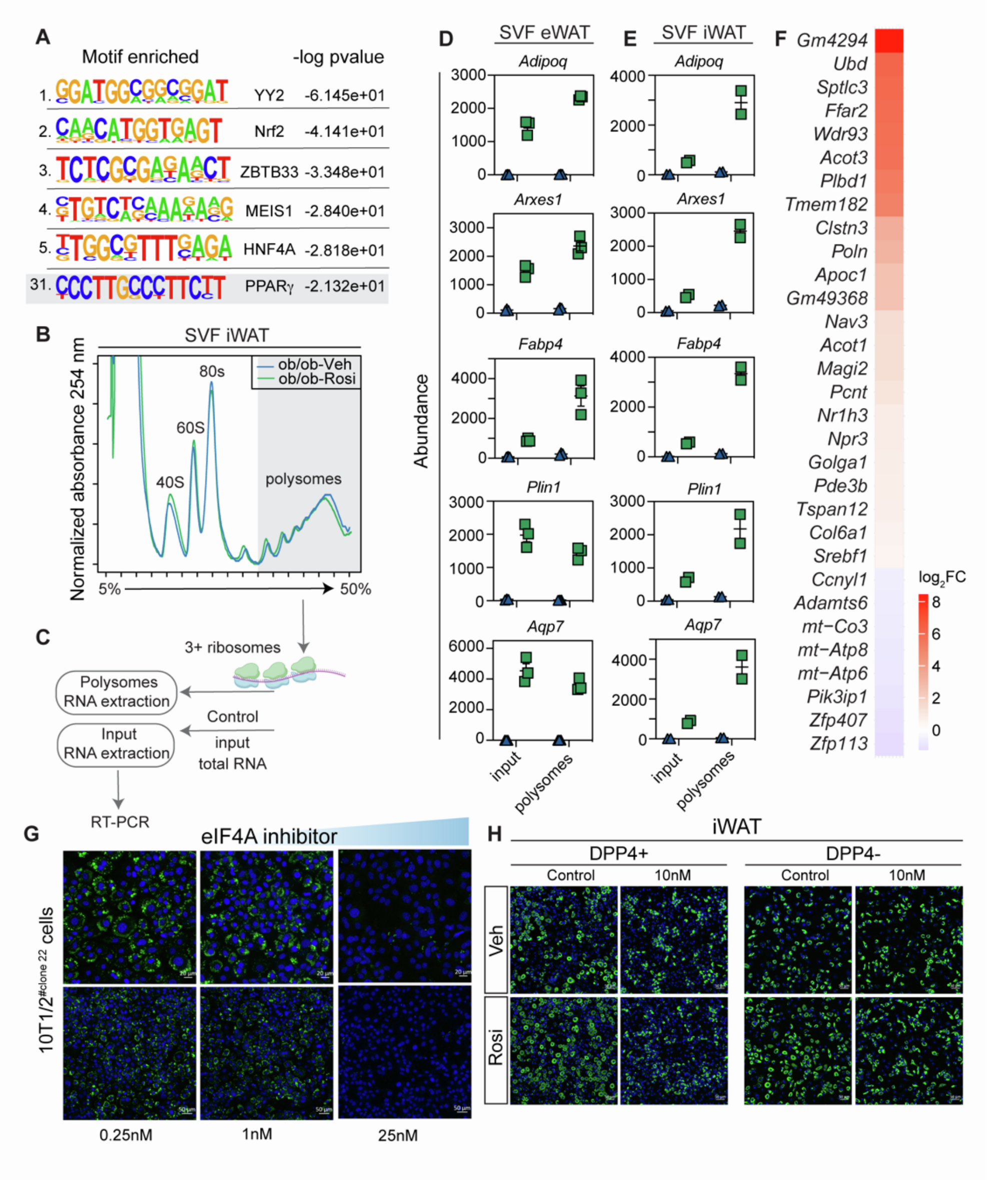

Fig.S10| Adipose-specific polysome profile. (**A**) Sequence motif enriched in the promoter regions of ribosome-related genes shown in Fig5A. (**B**) Polysome profile of primary stromal vascular fraction (SVF) of iWAT after four days of adipocyte differentiation. (**C**) Experimental design for the RT-PCR: fractions containing more than 3 ribosomes were pooled together for RNA extraction and cDNA synthesis. Control samples: input total RNA before samples were submitted to polysome fractionation. (**D-E**) Gene expression of *Adipoq, Arxes1, Fabp4, Plin1*, and *Aqp7* in the input (total RNA) vs polysomes in eWAT and iWAT (eWAT polysomes: triplicates, iWAT polysomes: duplicates). (**F**) Heatmap of 31 polysome fraction-unique DEGs affected by Rosi treatment in eWAT shown in Fig6F, ranked by log2FC value. (**G**) eIF4A inhibitor (CR-1-31-B) dose response in 10T/12 clone#22 cells. Cells were differentiated with DMI+GW for 1 day, and then treated for 48h with different concentration of CR-1-31-B. DAPI (nuclei- blue), LipidTox (neutral lipids- green). (**H**) ob/ob mice were treated with Veh or Rosi for 3 days, and SVF from iWAT was isolated. Cells were sorted as lineage negative (CD45-, CD31-), PDGFRα+, DPP4+, and DPP4-. Cells were kept in DMEM + insulin for 24 hours and then treated for 48h with eIF4A inhibitor to block translation. Confocal images: 2 wells per condition, 2 representative images per well were acquired.

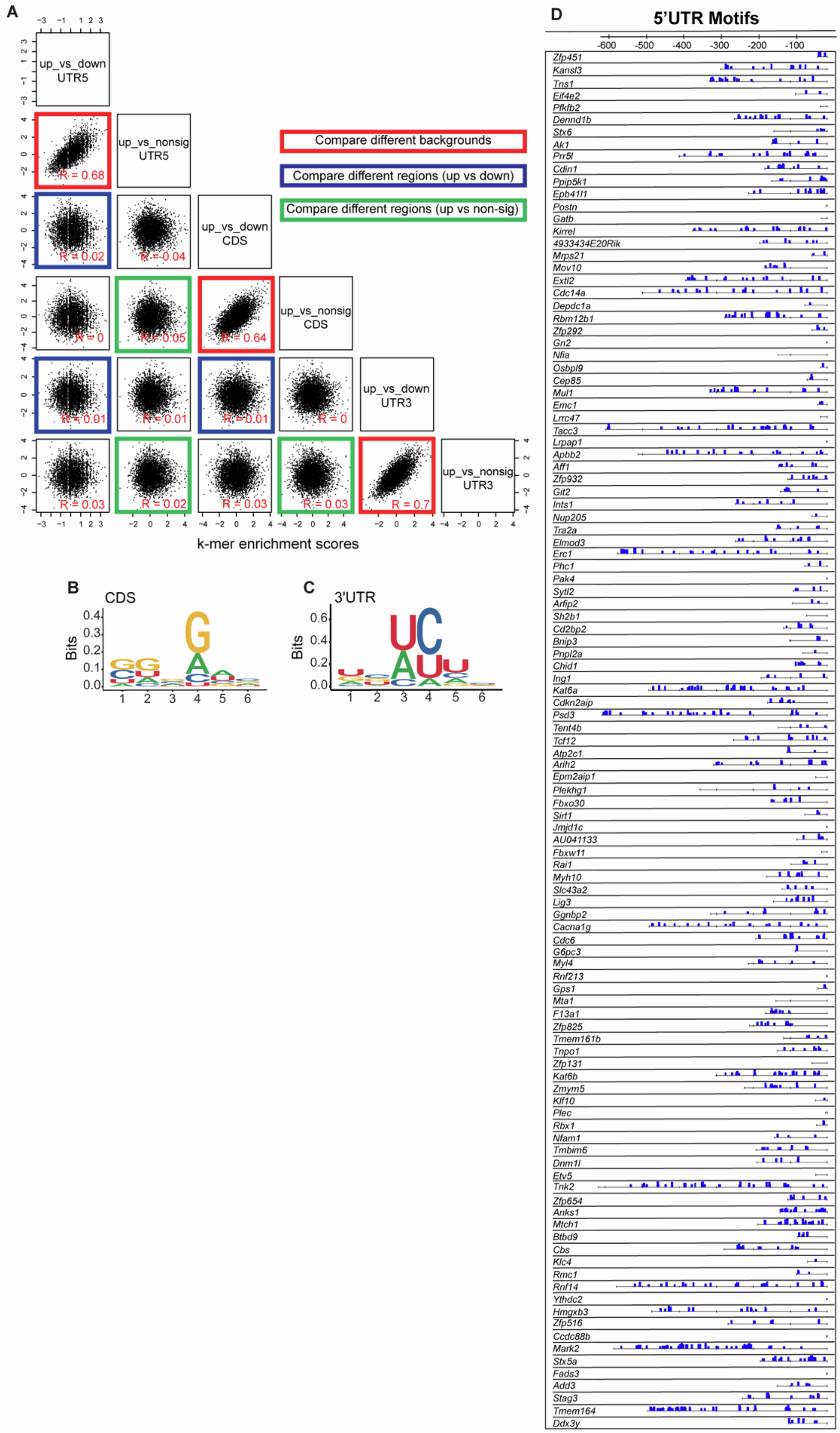
A
up_vs_down UTR5
up_vs_nonsig UTR5
up_vs_down CDS
up_vs_nonsig CDS
up_vs_down UTR3
up_vs_nonsig UTR3
R = 0.68
R = 0.02
R = 0.04
R = 0
R = 0.05
R = 0.64
R = 0.01
R = 0.01
R = 0.01
R = 0
R = 0.03
R = 0.02
R = 0.03
R = 0.03
R = 0.7
Compare different backgrounds
Compare different regions (up vs down)
Compare different regions (up vs non-sig)
k-mer enrichment scores
B
CDS
Bits
C
3'UTR
Bits
D
5'UTR Motifs
Zfp451
Kansl3
Tns1
Eif4e2
Pfkfb2
Dennd1b
Stx6
Ak1
Prr5l
Cdin1
Ppip5k1
Epb41l1
Postn
Gatb
Kirrel
4933434E20Rik
Mrps21
Mov10
Extl2
Cdc14a
Depdc1a
Rbm12b1
Zfp292
Gn2
Nfia
Osbpl9
Cep85
Mul1
Emc1
Lrrc47
Tacc3
Lrpap1
Apbb2
Aff1
Zfp932
Git2
Ints1
Nup205
Tra2a
Elmod3
Erc1
Phc1
Pak4
Sytl2
Arfip2
Sh2b1
Cd2bp2
Bnip3
Pnpl2a
Chid1
Ing1
Kat6a
Cdkn2aip
Psd3
Tent4b
Tcf12
Atp2c1
Arih2
Epm2aip1
Plekhg1
Fbxo30
Sirt1
Jmjd1c
AU041133
Fbxw11
Rai1
Myh10
Slc43a2
Lig3
Ggnbp2
Cacna1g
Cdc6
G6pc3
Myl4
Rnf213
Gps1
Mta1
F13a1
Zfp825
Tmem161b
Tnpo1
Zfp131
Kat6b
Zmym5
Klf10
Plec
Rbx1
Nfam1
Tmbim6
Dnm1l
Etv5
Trrk2
Zfp654
Anks1
Mtch1
Btbd9
Cbs
Klc4
Rmc1
Rnf14
Ythdc2
Hmgxb3
Zfp516
Ccdc88b
Mark2
Stx5a
Fads3
Add3
Stag3
Tmem164
Ddx3y

Fig. S11| Motif analysis of the 5'UTR of up-regulated transcripts under the regulation of Rosiglitazone. (**A**) The correlations of the hexamers enrichment scores between different regions (i.e., 5'UTR, CDS, and 3'UTR) or background choices regions (i.e., down-regulated transcripts and non-significantly expressed transcripts). The scatter plots in red squares highlight the correlations between different background choices during motif enrichment analysis (the X-axis represents the enrichment z-scores calculated using down-regulated transcripts as background, while the Y-axis represents the enrichment z-scores calculated using non-significantly expressed transcripts as background). The scatter plots in green squares highlight the correlations between different regions, the hexamer enrichment z-scores of which are calculated using non-significantly expressed transcripts as background. The scatter plots in blue squares highlight the correlations between different regions, the hexamer enrichment z-scores of which are calculated using down-regulated transcripts as background. (**B**) Sequence motif enriched in the CDS of up-regulated transcripts. (**C**) Sequence motif enriched in the 3'UTR of up-regulated transcripts. (**D**) The feature map of the 5'UTR in all up-regulated transcripts. Each deep blue bar indicates each significant site under the p-value lower than 0.05 calculated by the scan-matrix program.

Gene	Sequence forward	Sequence reverse
Adiponectin	CCGGAACCCCTGGCAG	CTGAACGCTGAGCGATACACA
Arxes1	ACGTGCTGTAACAATTTATGTGTCG	GCTTTACACGAGGTGAACAAACG
Fabp4	ACACCGAGATTTCCTTCAAACTG	CCATCTAGGGTTATGATGCTCTTCA
Plin1	AACGTGGTAGACACTGTGGTACA	TCTCGGAATTCGCTCTCG
Aqp7	AATATGGTGCGAGAGTTTCTGG	ACCCAAGTTGACACCGAGATA
Pdgfra	AGAAAATCCGATACCCGGAG	AGAGGAGGAGCTTGAGGGAG
Ucp1	GGCCTCTACGACTCAGTCCA	TAAGCCGGCTGAGATCTTGT
Cidea	ATCACAACTGGCCTGGTTACG	TACTACCCGGTGTCCATTTCT
Elovl3	TTCTCACGCGGGTTAAAAATGG	GAGCAACAGATAGACGACCAC
Dio2	CAGTGTGGTGCACGTCTCCAATC	TGAACCAAAGTTGACCACCAG
Cox8b	CCAGCCAAAACTCCCACTT	GAACCATGAAGCCAACGAC
Pgc1a	CCCTGCCATTGTTAAGACC	TGCTGCTGTTCCTGTTTTC
36B4	GGCCCTGCACTCTCGCTTTC	TGCCAGGACGCGCTTGT
Agpat2_PPRE	AGTCCTCAGACCACCTGACC	TCCAGTCCTGTCCTTCTCCA
Rpl11_PPRE eWAT	CCCTGCAAGAATACTTTGCCC	CAAGGGCCTTAGGGAACCAC
Rpl23a_PPRE eWAT	ACTGTAATATGAGGCTTCTTGGGA	CTTTCAAAGGGAAAGGTGGCG
Rps3a1_PPRE eWAT	CCTCGGGTGTAAAACGGGAA	TAGTCAGCACGTGAACAGCG
Rps27a_PPRE eWAT	AGCTTTGTCAGAATAAAAGCCCC	GGGCACACATCGGAGTAGTAG
Rps3a_PPRE BAT	CCTCGGGTGTAAAACGGGAA	TAGTCAGCACGTGAACAGCG
Rpl11_PPRE iWAT	TAGTGGACTGTCCTCGAAACT	GAGACTGTTCTTGGAGTCATGG
Rpl23a_PPRE iWAT	GCACTGATTGGATGAACTGTAATATG	GGTCTCCCACTCTAGGGAATTA

Supplemental Table 2. Primer sequences.
